# The programmed DNA elimination and formation of micronuclei in germ line cells of the natural hybridogenetic water frog *Pelophylax esculentus*

**DOI:** 10.1038/s41598-018-26168-z

**Published:** 2018-05-18

**Authors:** Magdalena Chmielewska, Dmitry Dedukh, Katarzyna Haczkiewicz, Beata Rozenblut-Kościsty, Mikołaj Kaźmierczak, Krzysztof Kolenda, Ewa Serwa, Agnieszka Pietras-Lebioda, Alla Krasikova, Maria Ogielska

**Affiliations:** 1 Department of Evolutionary Biology and Conservation of Vertebrates, Faculty of Biological Sciences, University of Wrocław, Sienkiewicza 21, 50-335 Wrocław, Poland; 2 Department of Cytology and Histology, Faculty of Biological Sciences, Saint-Petersburg State University, 7/9 Universitetskaya emb., 199034 St. Petersburg, Russia; 3 Department of Human Morphology and Embryology, Division of Histology and Embryology, Wrocław Medical University, Chałubińskiego 6a, 50-368 Wrocław, Poland; 4 Laboratory of DNA Analysis, Faculty of Biological Sciences, University of Wrocław, Sienkiewicza 21, 50-335 Wrocław, Poland

## Abstract

DNA elimination is a radical form of gene silencing and occurs both in somatic and germ cells. The programmed DNA elimination occurs during gametogenesis in interspecies hybrids that reproduce by hybridogenesis (stick insects, fishes, and amphibians) and concerns removal of whole genomes of one of the parental species and production of clonal gametes propagating the genome of the other species. The cellular mechanisms differ considerably in hybridogenetic insects and fishes but remains unknown in edible frogs *Pelophylax esculentus*, natural hybrids between *Pelophylax lessonae* and *Pelophylax ridibundus*. Here we report DNA elimination mechanism in early developing gonads of diploid and triploid hybrid frogs, studied by TEM, immunofluorescence, and cytochemistry. In gonocytes of both sexes (primary oogonia and prespermatogonia), micronuclei emerge as detached nuclear buds formed during interphase. We found depletion of nuclear pore complexes in micronuclear membrane and chromatin inactivation via heterochromatinization followed by degradation of micronuclei by autophagy. Micronuclei formation does not lead to apoptotic cell death showing that genome elimination is a physiological process. Chromatin elimination via micronuclei in *P. esculentus* is unique among hybridogenetic animals and contributes to broadening the knowledge about reproductive modes in animals.

## Introduction

The physiological programmed DNA elimination, known also as chromatin diminution or chromosome elimination, is a radical form of gene silencing and has been described in more than a hundred animal species from nine taxonomic groups, ranging from Protista (ciliates), invertebrates (nematodes, copepods, insects, arachnids) to vertebrates (lampreys, hagfish, birds, and marsupials)^1,2^. The amount of the eliminated genomic DNA ranges from about 20% in the hagfish and nematodes to 95% in ciliates. In invertebrates, programmed DNA elimination occurs during early embryonic development and concerns both somatic^1,2^ and germline cells^3,4^. In the latter case, DNA is eliminated from early gonocytes (primary oogonia or prespermatogonia) or during the first meiotic division of spermatogenesis and concerns determination of the male sex^5–8^. Similar mechanism was also described in a cartilagous fish *Hydrolagus colliei*^3^ and in a passerine bird, the zebra finch^4,5^. Genome elimination from germline cells occurs also during sporogenesis in plant hybrids between *Brachiaria ruziziensis* and *B. brizantha*^6^.

A peculiar kind of programmed DNA elimination occurs in interspecies hybrids that reproduce by hybridogenesis and concerns removal of whole genomes of one of the parental species. The hybrids are restored in each generation by mating with a species whose genome was eliminated from the germline^7^. Hybridogenesis has emerged independently in a few evolutionary distant animal groups (insects, fishes, amphibians). Although its result is the same, i.e. elimination of one of the parental genomes from the germline cells and clonal propagation of the other one, the cellular mechanisms differ considerably. Elimination of genomes may occur pre-meiotically during the phase of gonocyte proliferation or during meiosis. This specific process was originally described in a female hybrid fish *Poeciliopsis monacha-lucida* (Cyprinidae)^8^ but later was also found in other interspecies hybrid fishes from the genus *Hypseleotris*^9^ and *Hexagrammos*^10^, as well as in the female hybrid stick insects (Phasmodea, Insecta)^11–14^. Genome elimination was also found in a variety of triploid hybrid organisms: among fishes *Squalius alburnoides*^15^, *Phoxinus eos-neoagaeus*^16^ and *Misgurnus anguilicaudatus* complex (Cobitidae)^17–20^; amphibians: the Batura toads of *Bufotes viridis* group^21^ and the edible frog *Pelophylax esculentus* (formerly *Rana esculenta*)^22,23^.

Hybridogenesis has also been discovered in the western Palearctic water frogs of the *Pelophylax esculentus* complex^24,25^. *Pelophylax lessonae* (LL) and *P. ridibundus* (RR) are diploid species, whereas *P. esculentus* is their interspecific hybrid represented by diploid (RL) or triploid (RRL, LLR), and occasionally non-viable tetraploid (RRLL) and pentaploid (RRLLL) males and females that usually coexist with one of the parental species in the same populations^26–29^. The hybrids eliminate one of the parental genomes from their germline cells before meiotic recombination, duplicate the remaining one and undergo meiosis; however, the recombination occurs between the identical copies of the duplicated chromosomes, as was evidenced in diplotene oocytes^30–32^. As the result, the retained genome is transmitted clonally and F1 hybrids are restored *de novo* in each generation when mating with this parental species, whose genome was eliminated^33,34^. The retention and duplication of one of the genomes (R; *ridibundus*) was first detected by Tunner and Heppich^35^ and Tunner and Heppich-Tunner^36^ in juvenile hybrid females.

Cytogenetic mechanisms of the programmed DNA elimination remain poorly understood in the majority of cases with exception of the ciliate protozoa^37^. Chromosome elimination in insects and birds (finches) involves a wide spectrum of epigenetic modifications including heterochromatinization, histone H3/H4 acetylation, H3S10 phosphorylation, and DNA methylation^38,39^. Ravi and Chan^40^ and Sanei *et al*.^41^ found that the mechanism underlying haploidization in plants is also connected with changes of histone H3 in centromeric regions of chromosomes that compromises the assembly site for the kinetochore complex. Similar processes are involved in unipolar spindle formation and monocentric cell divisions during spermatogenesis in insects *Mycophila speyeri*^42^, *Sciara coprophila*^43,44^, and fishes *Poeciliopsis monacha-lucida*^45^.

A different mechanism involves formation of micronuclei and concerns both the programmed and non-programmed DNA elimination. Micronuclei are specific extranuclear structures morphologically similar to the main cell nucleus, surrounded by the double membrane, and containing the eliminated DNA. They are usually formed during aberrant mitoses displaying chromosome missegregation and contain chromosomes that are not bound to the spindle and lag behind during anaphase and telophase^46–50^. Micronuclei may also arise at interphase as the result of nuclear budding^47–49,51^. However, the fate of encapsulated chromatin remains unknown in a majority of cases. Micronuclei were observed in somatic cells of plant hybrids^49^, in male germline cells in the zebra finch^4,5^, and in somatic cells of the sea lamprey^52^. Moreover, they are well-known structures that appear as a result of chromosome missegregation and reflect chromosome instability in many kinds of cancer cells^47^ and a variety of cells exposed to genotoxic agents^53^.

Micronuclei (described formerly as *nucleus-like bodies*, NLB^54^) were also observed in the germline cells of developing gonads in *Pelophylax esculentus*^34,54,55^, however the cytological mechanisms of their appearance remained unknown. From the earliest stages of differentiation, tadpole gonads are composed of somatic cells (pre-follicular in the ovaries and pre-Sertoli in the testes) and germline cells. Germ cells that arise from primordial germ cells of an embryo are gonocytes, which proliferate before and after sexual differentiation of gonads, eventually giving rise to the limited pools of primary oogonia in females^56–58^ and prespermatogonia in males^58^. These events take place only during larval and juvenile periods of individuals’ lives. Prespermatogonia are the only germ cells in a male gonad before sexual maturation and afterwards they transform into spermatogonial stem cells^59^. The situation is different in females where meiosis starts early, so in developing ovaries we can observe both mitotically dividing gonocytes and meiocytes up to early diplotene that form a limited stockpile for the entire female’s life (for details see Ogielska *et al*.^60^). Meiocytes (but not gonocytes) in amphibians form isogenic groups and are encapsulated inside cysts; only in females diplotene oocytes are individual cells (each inside its own ovarian follicle). Because of the large variety of names used in the literature, we decided to apply a general name “gonocytes” for female and male germline cells during the phase of mitotic multiplication before formation of cysts and meiotic entry in larval stages. Nuclei of gonocytes in *Pelophylax*, as in other ranid frogs, are spherical and smooth in outline in contrary to most amphibian species studied so far, where nuclei are highly lobulated^56,61^.

In the present paper we provide results of the first complete examination of micronuclei in the gonocytes of diploid and triploid water frog hybrids. We focused on ultrastructure, spatial relationship with gonocyte nuclei, time of appearance, and presumed mechanisms of micronuclei formation and their fate. The aims of the study were to check whether micronuclei in *P. esculentus* undergo epigenetic chromatin modifications and alterations of nuclear envelope. We consider that micronucleation is a physiological process, which does not lead to gonocyte death, whereas micronuclei, as carriers of the eliminated DNA, are directed to autophagic process while the main nuclei remain intact.

## Results

### Morphology (TEM and fluorescence microscopy)

**Figure 1:**
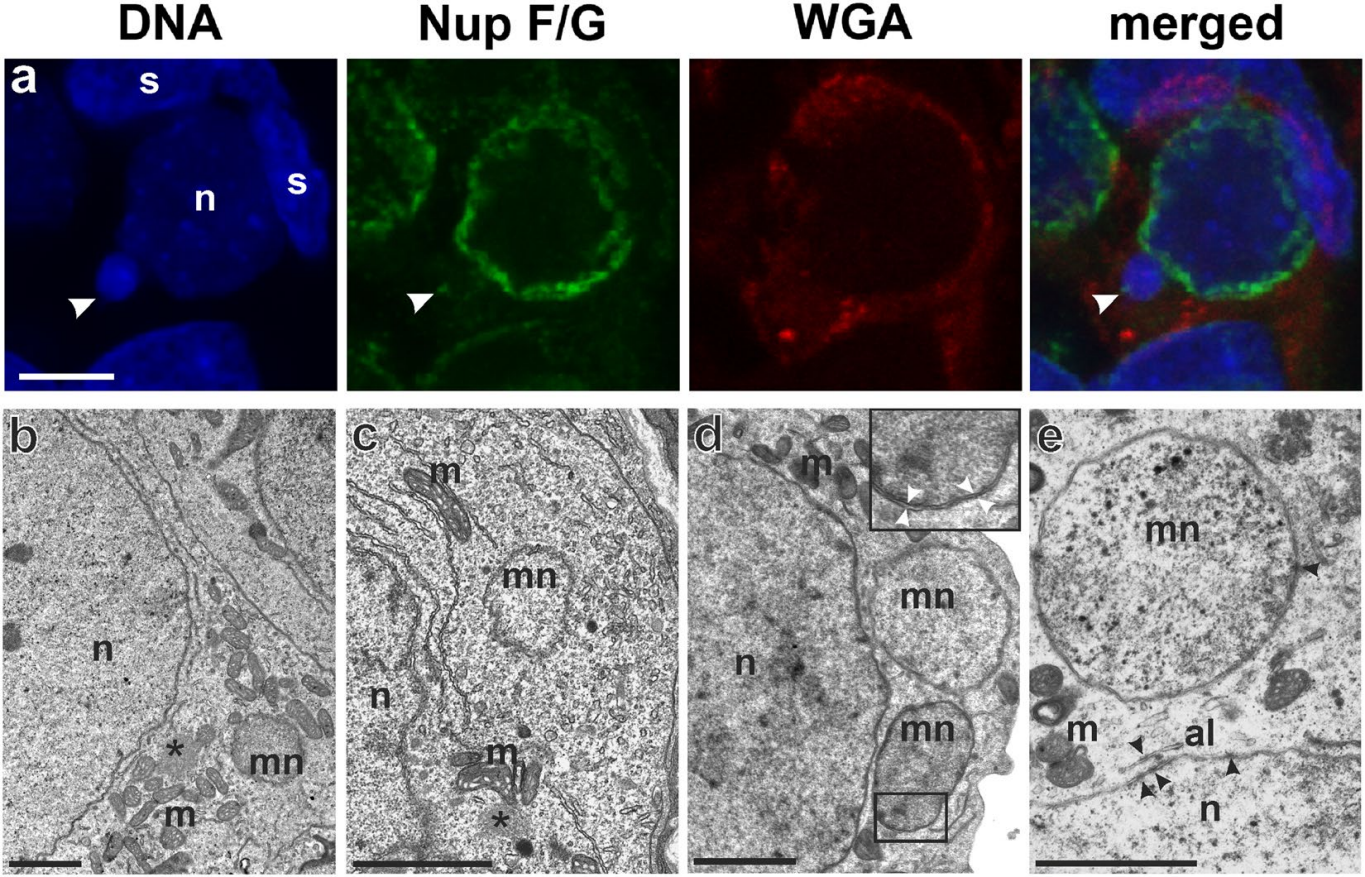
Morphology of the micronuclei in gonocytes of *P. esculentus*. (**a**) Immunofluorescent staining of frozen tissue sections of testis from male at 28 Gosner stage: cell membrane (wheat germ agglutinin, WGA, red) and nuclear pore complex proteins, NPC (Nup F/G, green), DNA counterstained with DAPI (blue). Confocal image represents projection of 4 z-sections (0.46 µm thick) to highlight the weak NPC staining at the rim of micronucleus (arrowhead) contained inside the cell boundaries probed with WGA. Note the weaker chromatin signal in nuclei of germ cells (n), corresponding to euchromatin, and stronger signal in nuclei of somatic cells (s), corresponding to heterochromatin. (**b–e**) TEM images of micronuclei present in prespermatogonia (**b,c**) and gonocytes (**d,e**). Micronuclei were found in both diploid (**b,d,e**) and triploid (**c**) hybrids during the premetamorphosis stage of somatic development according to Gosner (1960), stage 36 (**d,e**) or during metamorphosis climax, stage 46 (**b,c**). Double membrane envelope was surrounding chromatin in micronuclei, similarly as in main nuclei (inset in **d**). Chromatin condensation level differed from euchromatin (**c–e**) to heterochromatin (**b,d**). Cytoplasm of germ cells was filled with membranes (al), mitochondria and nuage material (asterisk) was present in the vicinity of cell nucleus. (**e**) Note the nuclear pore complexes (arrowhead) abundant in nuclear envelope of the main nucleus and annulate lamellae but rare in micronuclei. al- annulate lamellae, n – nucleus, m – mitochondria, mn – micronucleus, s – somatic cells. Scale bars (**a**) 5 µm, (**d-e**) 2 µm.

The difference between size and morphology of somatic and germ cells was evident in tadpole gonads. Somatic cells had pyramidal nuclei with clearly visible heterochromatin patterns, whereas interphase gonocytes had bigger spherical nuclei, 10–15 µm in diameter, with smooth outline and euchromatin content (Fig. 1a). Most gonocytes were in interphase and all encountered mitoses had regular distribution of segregating chromosomes.

**Figure 2:**
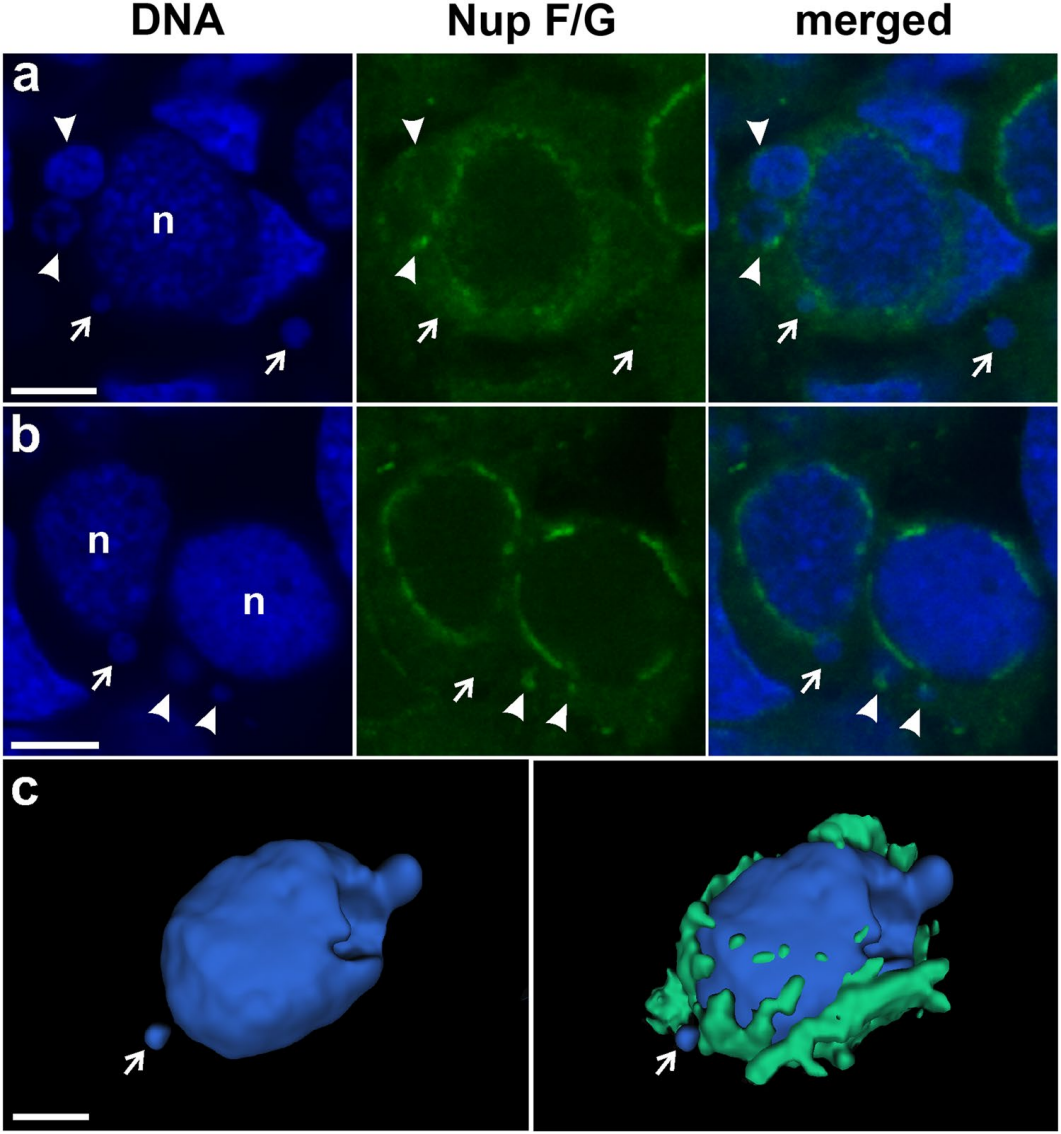
Level of nuclear pore complex proteins is reduced in nuclear envelopes of micronuclei in contrast to the main nucleus. Immunofluorescent staining of frozen tissue sections of ovaries from *P. esculentus* diploid female at 29 Gosner stage: nuclear pore complex proteins, NPC (Nup F/G, green), DNA counterstained with DAPI (blue). Germ cells in the centre of the images (n) have higher expression level of NPC proteins in the nuclear envelope in contrast to somatic cells, visible in the surrounding tissue. The NPC proteins staining pattern in MN is patchy, dotted (arrowhead) or absent (arrow). (**a**) Gonocyte with 3 micronuclei of various sizes. Micronucleus in the middle has patchy NPC proteins staining similar to main nucleus. (**b**) On the left side is gonocyte with MN devoid of NPC proteins (arrow), on the right side gonocyte with dotted NPC proteins signals in two MN of various sizes (arrowheads). Confocal images represent projection of 2 z-sections (**a**) or single z-section (**b**) (0.46 µm). (**c**) 3D model of reconstructed fluorescent signal volumes from cell visible in Fig. 9d, NPC proteins (green) and DNA (blue). Note that NPC signal is discontinuous probably due to differential distribution of nuclear pore complexes in the nuclear envelope and threshold settings excluding cytoplasmic signal. Scale bars 5 µm.

In cytoplasm of gonocytes of hybrid tadpoles, both diploid (RL) and triploid (RRL and LLR) we observed chromatin-positive bodies, i.e. micronuclei (Fig. 1a). Micronuclei were small spherical structures morphologically resembling the main nucleus (Figs 1b–e, 2a,b) and containing DNA, as confirmed by DAPI staining (Fig. 1a DAPI). They had the double membrane envelope that resembled that of the main nucleus but was almost depleted of the nuclear pore complexes (Fig. 1d,e). The cytoplasm of gonocytes contained intracellular membranes (endoplasmic reticulum, annulate lammelae) lying close to the nuclear envelope (Fig. 1b,c,e). Micronuclei differed in size (0.8–4 μm) and number (1–5) per one cell, and were localized at various distances from the main interphase nucleus (Fig. 1b–e), as confirmed in 3D reconstructions from confocal images (Fig. 2c). The size and number of micronuclei per one cell was analysed in various tadpole developmental stages from sexual differentiation until completion of metamorphosis (Gosner^62^ stages 28–46) and genomic composition (RL, RRL, RLL). In total number of 907 micronuclei analysed most frequently found were micronuclei of diameter 1.1–2 and 2.1–3 μm (37.82 and 38.26%, respectively), the smallest were 0.8–1 μm (3.42%), and the biggest were 3.1–4 μm (20.5%) (see Supplementary Fig. 1 and Supplementary Table 1). The prevailing group of cells possessed one micronucleus (67.2% of all cells), two micronuclei were found inside smaller group of cells (21.67%), three micronuclei were present in 9.08%, and four and five micronuclei were rarely seen (1.76% and 0.29%, respectively of total 683 cells) (see Supplementary Fig. 2 and Supplementary Table 2). Plasma membrane staining using wheat germ agglutinin (WGA) confirmed the presence of micronuclei inside the cytoplasm of gonocytes in both sexes (Fig. 1a). Frequency of micronucleated gonocytes in diploid and triploid hybrids was recently reported and ranged from 15 to 35% (see^55^); whereas in gonads of the parental species we have not found any cellular structures similar to micronuclei.

### Nuclear envelope antigens in micronuclei

**Figure 3:**
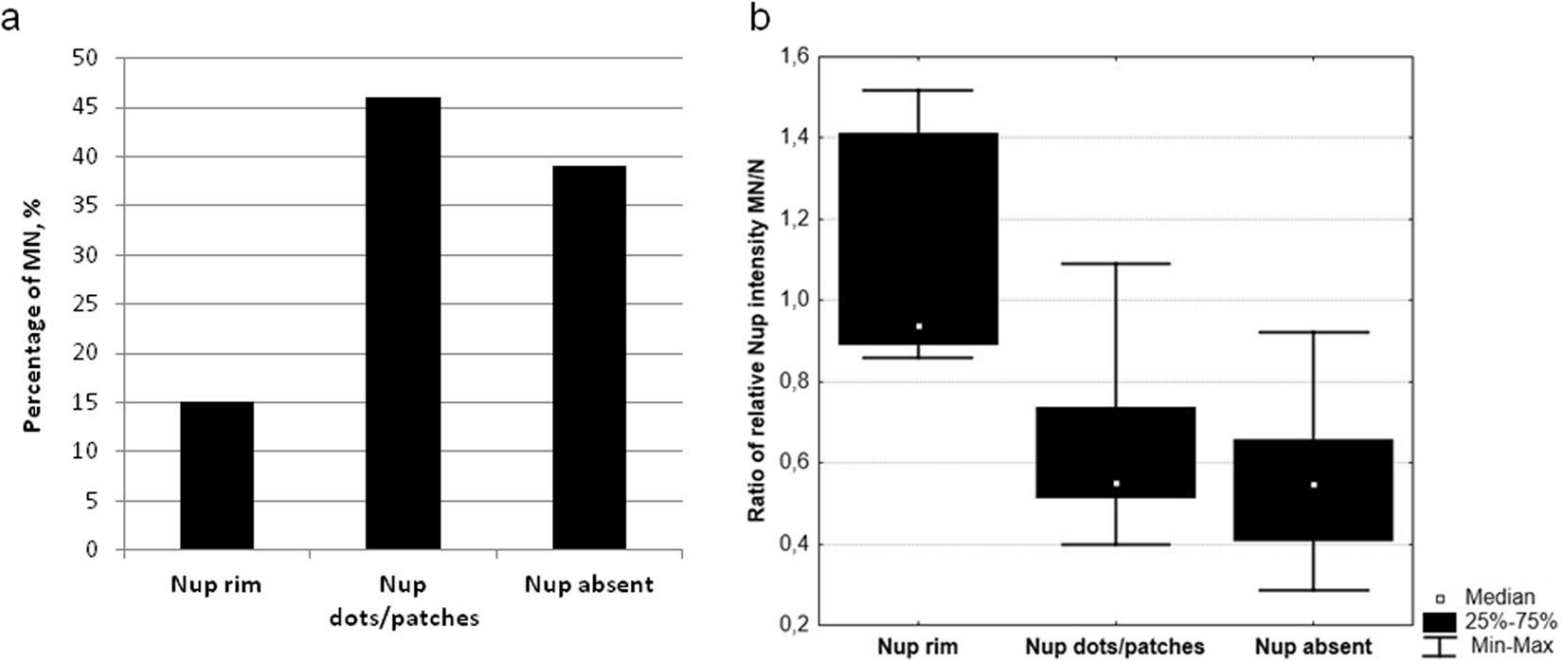
Quantitative analysis of nuclear pore complex proteins in micronuclei. (**a**) Frequency of micronuclei (MN) with various distribution of nuclear pore complex proteins (Nup) assessed by morphological criteria. (**b**) Ratios of Nup relative fluorescence of micronuclei (MN) to main nuclei (N) according to three classes of Nup distribution (rim, dots/patches, absent) in gonocytes of hybrid frogs.

The TEM examination of the nuclear envelope revealed the reduced number of the nuclear pore complexes (NPC) in the micronuclei (Fig. 1e - arrowheads). We examined the density and distribution of NPC using antibody reacting with a conserved domain of FG-repeat nucleoporins. NPC protein signals were detected as dots or patches in the nuclear envelopes of nuclei of somatic cells and gonocytes in ovaries and testes of all examined individuals (Figs 1a and 2). Interestingly, gonocytes revealed much higher expression level of NPC proteins comparing to somatic cells, as well as the presence of cytoplasmic signal, which was absent in somatic cells (Fig. 2). The same staining pattern was observed in *P. lessonae* (Supplementary Fig. 3) and *P. ridibundus* (not shown), as well as in hybrid gonads (Figs 1, 2). A portion of gonocytes displayed micronuclei surrounded by NE with similar or lower staining level of FG-repeat nucleoporins as compared to the main nucleus (Fig. 2a - arrowhead, lower micronucleus). Micronuclei with condensed heterochromatic DNA displayed lower expression level of NPC proteins than in nuclear envelope of the main nucleus. Among 106 micronuclei analysed in 80 cells, FG-repeat nucleoporins were detected in 61% as patches (Fig. 1a) or dots on the chromatin rim (Fig. 2b - arrowheads). We found that 15% of micronuclei showed the rim staining of entire envelope and 46% possessed only 1–3 signals, while the signal was absent in 39% of micronuclei (Fig. 2a,b - arrows, Fig. 3a). Significant portion of gonocytes displayed at the same time micronuclei with remnants of nucleoporins together with nucleoporins-negative ones (Fig. 2a). We have supported morphological observations by quantitative densitometric analysis of relative fluorescence signal for NPC proteins present in nuclear envelopes (ratio of mean fluorescence values of micronucleus to main nucleus). The ratios were significantly different in the three groups of micronuclei, classified according to morphological NPC proteins expression pattern. The highest values were found in micronuclei possessing rim signal of entire envelope, lower values were noted in micronuclei having dots and patches, and the lowest values in those without nuclear envelope signal (Fig. 3b, Supplementary Table S3). Additionally our analysis showed that in the majority of MN the mean ratio value was 0.64 or lower, revealing depletion of NPC from micronuclear envelope (Supplementary Table S3). The results of TEM and NPC proteins level analyses indicated that the envelopes of micronuclei were gradually destroyed, which most probably affected its function.

### Histone modifications of chromatin in micronuclei

**Figure 4:**
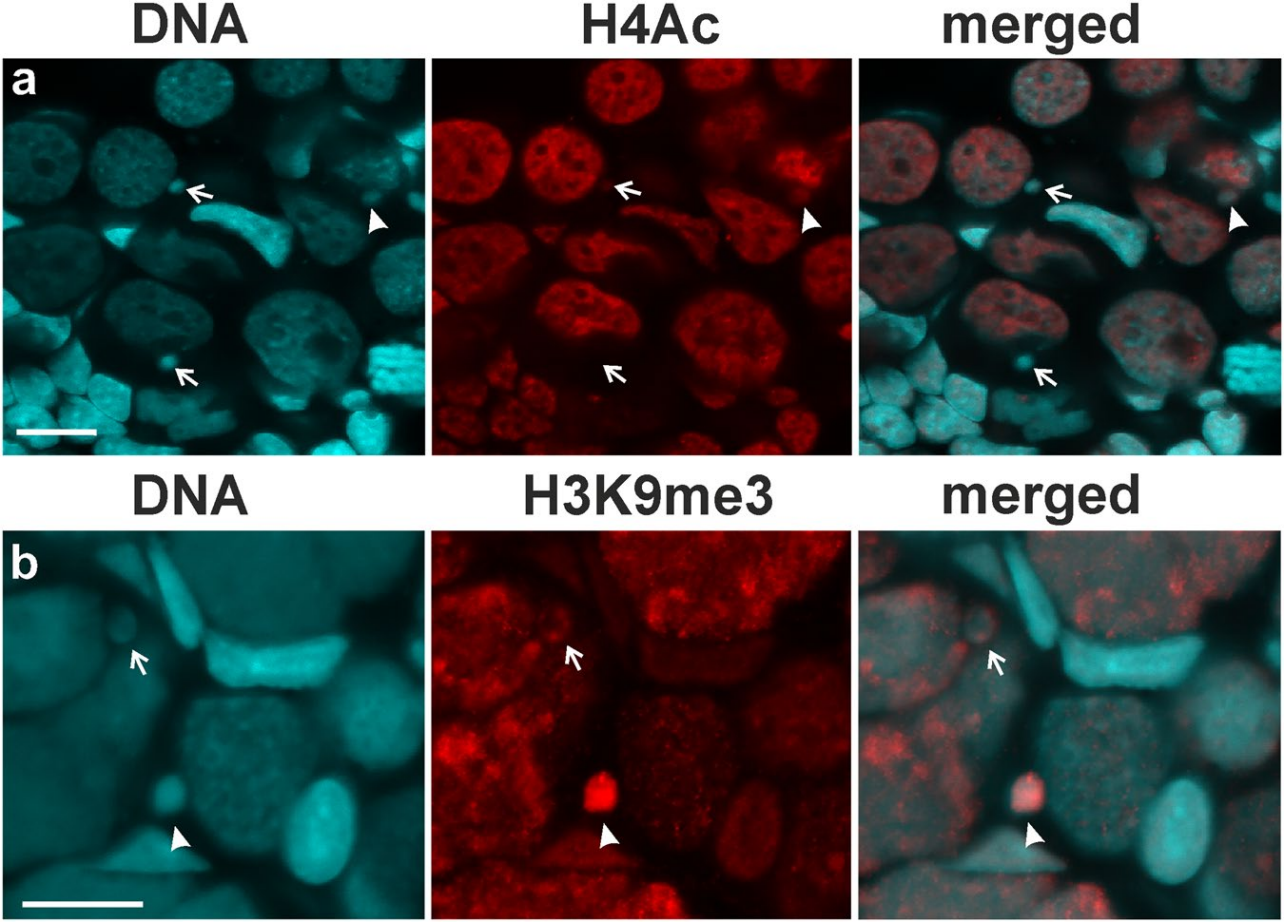
Chromatin in the micronuclei in contrast to gonocytes nuclei is inactivated and accumulates heterochromatin modification. 3D imunofluorescence of whole mount gonads of diploid *P. esculentus* females at 32 Gosner stage: (**a**) Chromatin in the micronuclei, but not in the gonocytes nuclei, revealed decreased level (arrow, upper micronucleus) or even absence (arrow, lower micronucleus) of acetylated histone Н4, the modification corresponding to active chromatin. Some micronuclei still retain level of H4Ac similar to main nucleus (arrowhead). (**b**) Chromatin in the micronuclei accumulates H3K9me3 modification indicating its inactivation and heterochromatinization (arrowhead). Some micronuclei still retain level of H3K9me3 similar to main nucleus (arrow). Images represent single z-section of the gonad. Scale bars 10 µm.

Hybrid gonocytes were harbouring micronuclei, in which chromatin was at various stages of condensation (Fig. 1b-e), either more condensed than in the main nucleus (Figs 1a, 4a - arrows, B - arrowhead) or at similar condensation level (Fig. 2b - arrow). Morphological observations were in agreement with densitometry analysis of fluorescence intensity after DAPI staining. Similar condensation level of chromatin was observed in 51% of micronuclei, 43% showed higher condensation, and 6% displayed lower signal (Supplementary Fig. S4), as compared to main nuclei. For more detailed characterization of chromatin state (eu- or heterochromatic) we performed two indirect approaches to show immunofluorescent staining of epigenetic modifications of histones: (1) against acetyl-histone H4 (H4Ac) as a marker of transcriptionally active chromatin (euchromatin), and (2) against histone H3 tri-methylated at lysine 9 (H3K9me3) as a marker of heterochromatin. Chromatin modification pattern in the main nuclei of gonocytes was similar both in the parental species and in the hybrids. Most of the interphase nuclei of gonocytes were euchromatic, as was detected with antibodies against H4Ac (Fig. 4a). On the contrary, we detected a reduced level of the H4Ac staining in all observed micronuclei. As detected by immunofluorescence intensity analysis, 42% (36 micronuclei) exhibited reduction of the intensity of H4Ac signal from 0% to 50% comparatively to main nuclei (Fig. 4a - upper arrow and arrowhead, Supplementary Fig. S5); 53% (46 micronuclei) showed reduction of signal from 50% to 100% comparatively to main nuclei, while 5% (4 micronuclei) exhibited reduction of signal higher than 100% comparatively to main nuclei (Fig. 4a - lower arrow, Supplementary Fig. S5). Heterochromatin detected with antibody against H3K9me3 histone mark was observed in compact chromatin regions of interphase nuclei of somatic and in some germ cells. A portion of micronuclei (50 micronuclei, i.e. 40%) revealed increased fluorescence signal after detection of H3K9me3 modification from 0.1% to 50% in comparison to main nuclei (Fig. 3b - arrow, Supplementary Fig. S6); 22% (28 micronuclei) showed increased signal from 50% to 100%, and 15% (19 micronuclei) showed more than 100% increase of the signal (Fig. 3b - arrowhead, Supplementary Fig. S6). Interestingly, we also found that 23% (19 micronuclei) demonstrated reduction of the H3K9me3 mark (see Supplementary Fig. S6). Thus, the results of epigenetic histone modification approaches are in agreement with both the ultrastructure observations and DAPI-positive compact staining in micronuclei in gonocytes of hybrid tadpoles (Figs 1 and 2). This in turn suggest that DNA present in micronuclei behaved differently than in the main nuclei and most probably became inactivated by heterochromatinization.

### Formation and degradation of micronuclei

**Figure 5:**
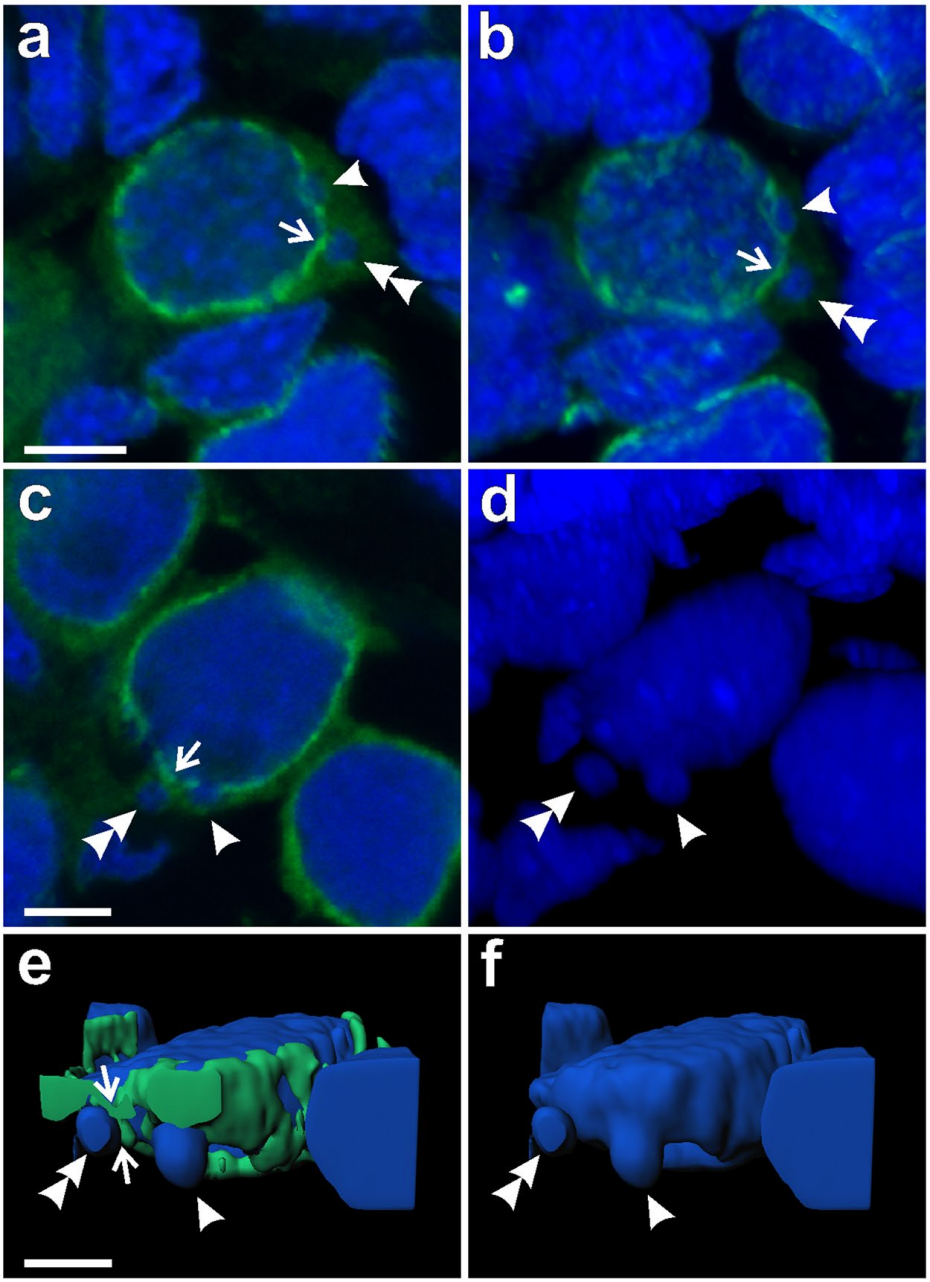
Formation of micronuclei from nuclear extrusions in gonocytes. Immunofluorescent staining of frozen tissue sections of ovaries from *P. esculentus* diploid females at 41 (**a,b**) and 44 (**c–f**) Gosner stages: nuclear pore complex proteins, NPC (Nup F/G, green), DNA counterstained with DAPI (blue). (**a–c**) Single confocal sections of DNA and NPC proteins (0.46 µm). (**b,d**) 3D reconstructions of natural confocal sections showing the views of germ cells from the angle to visualise chromatin budding (arrowheads). (**e,f**) 3D models of reconstructed fluorescent signal volumes from cell visible in **c,d**, (**e**) NPC proteins and DNA, note that NPC signal is discontinuous probably due to differential distribution of nuclear pore complexes in the nuclear envelope and threshold settings excluding cytoplasmic signal, (**f**) DNA. (**a,b**) Early stage of micronucleus formation: gonocyte with small chromatin extrusion possessing a weak NPC protein signal in nuclear envelope and strong DAPI signal (arrowhead), representing emerging micronucleus containing heterochromatin. Second micronucleus with punctate NPC protein signal (a, arrowhead). (**c,d,f**) Middle stage of micronucleus formation: extruded chromatin has finger-like shape and is connected with main chromatin mass in the cell nucleus (arrowhead). Note the discontinuous NPC signal in the nuclear envelope at budding site. Late stage: micronuclei (double arrowhead, a–e) are separated from the main nucleus by nuclear envelope with strong NPC protein signal (arrow), either protruding towards the micronucleus (**a**) or flat (c,e). There is no distinct NPC protein signal at the nuclear envelope in micronucleus (**c**) as well as in finger-like extrusion (arrow, c,e). Scale bars 5 µm.

To answer the question whether micronuclei detached from the main nuclei by budding, we analysed confocal microphotographs of gonocytes labelled for plasma membrane and NPC proteins (WGA and FG-repeat nucleoporins) in search of micronuclei situated in the closest vicinity of the main nuclei. In several interphase gonocytes we found nuclear blebs similar to micronuclei in shape and size protruding from the main nuclei (Fig. 5a,c - arrowheads), which we interpreted as micronuclei budding. We could distinguish an extruded DAPI-positive content of a forming micronucleus surrounded by nuclear envelope displaying a weak NPC protein signal (Fig. 5a,b - arrowhead, Supplementary Movie S1), representing an early stage of micronucleus formation. At the same time we could see an aggregation of Nup signal in nuclear membrane around the base of the budding chromatin (Fig. 5a,b - arrowhead). The next step of budding was illustrated by the finger-shaped chromatin extrusion tightly attached to the main nucleus (Fig. 5c–f - arrowhead, Supplementary Movie S2). Finally, micronuclei were separated from the main nucleus by NPC proteins-positive membrane protruding from the main nucleus toward the micronucleus (Fig. 5a,c,e - arrow). In the already separated micronuclei we observed a lower level (Fig. 5a - double arrowhead) or lack of NPC proteins (Fig. 5c - double arrowhead). This suggests that either during chromatin budding the nuclear membrane surrounding micronucleus was depleted of majority of NPC proteins or micronuclei are extruded from the main nuclei at nuclear envelope regions of low NPC proteins level.

**Figure 6:**
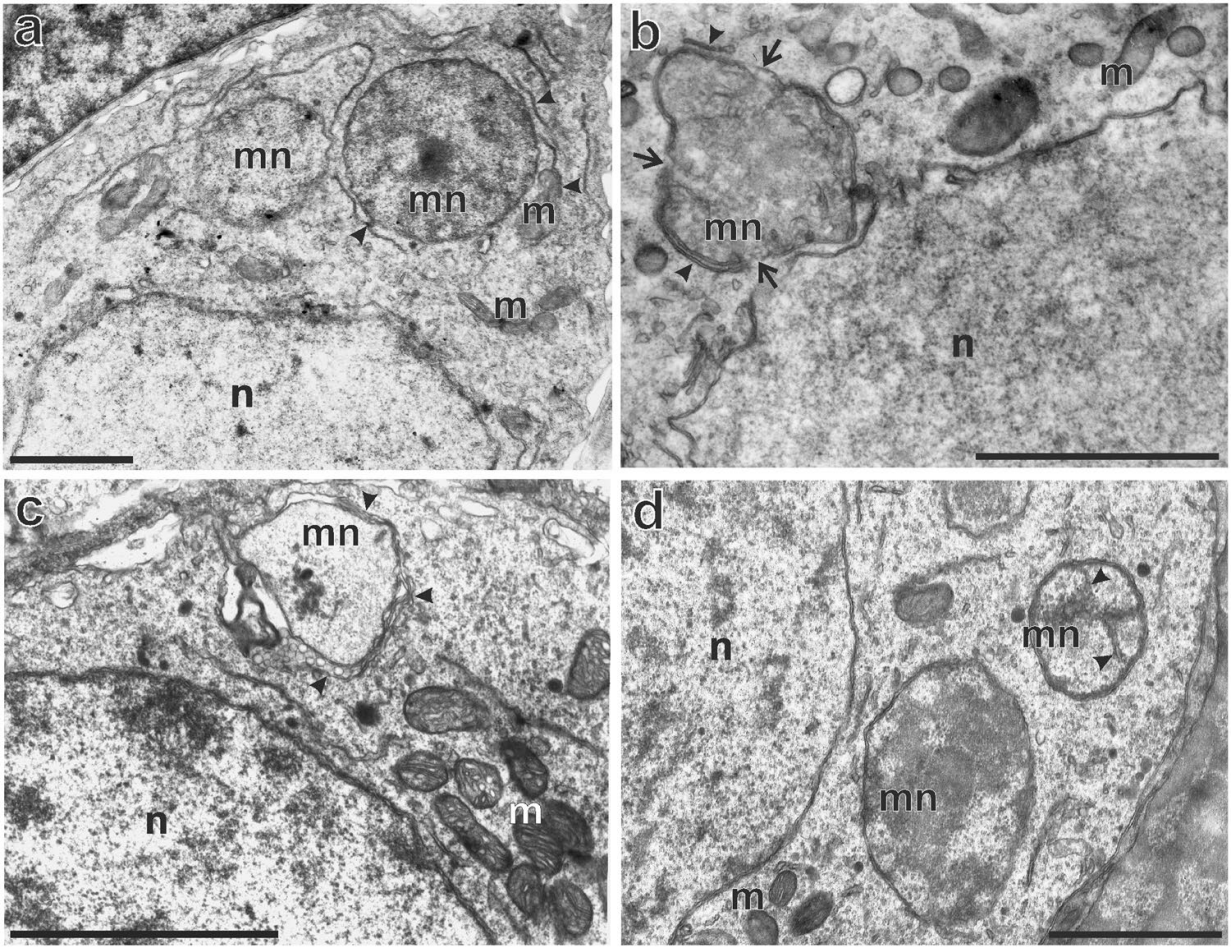
Chromatin in micronuclei is degraded by autophagy. Transmission electron microscopy analysis of autophagosome formation around micronuclei and invasion of tubular membranes inside the chromatin territory. Micronuclei were found in diploid hybrid males (**a**) and females (**b**–**d**) during the premetamorphosis stage of somatic development according to Gosner (1960), (**a**–**d**) stages 37, 41, 31, 36 respectively. (**a**) Sequestration of the cytoplasm by lipid double membranes (arrowheads) surrounding large heterochromatinized, intact micronucleus. (**b**) Micronucleus accompanied by two membranes assembling on the surface (arrowheads): cap-shaped (at the bottom) and linear (upper part), inside the chromatin the invading membranes are visible. Arrows point the disruptions of nuclear envelope in micronucleus (**c**) Autophagosome membranes (arrowheads) encapsulating micronucleus are partially double- or single layered. Vacuolar spaces are signs of degeneration (white spaces) and vesicles similar to lysosomes are visible. (**d**) Micronucleus with tubular membranes invading chromatin. n – nucleus, m – mitochondria, mn – micronucleus. Scale bars 2 µm.

To further investigate the fate of micronuclei we examined the gonocyte morphology in TEM images. Along with regular and intact micronuclei we also found micronuclei presenting various signs of degeneration. We observed sequestration of the cytoplasm by lipid double membranes, apparently endoplasmic reticulum and annulate lamellae, surrounding heterochromatinized intact micronuclei (Fig. 6a - arrowheads), which might reflect an initial stage of autophagy. We also found membranes assembling on the surface of micronucleus envelope (Fig. 6b - arrowheads) or encapsulating degenerating micronuclei (Fig. 6c - arrowheads) that we interpreted as autophagosomes (nucleophagosomes according to Mijaljica and Devenish^63^). The presence of vacuolar spaces, lysosomal vesicles (Fig. 6c), and disruption of micronucleus envelope (Fig. 6b - arrows) suggests the autophagic degradation of chromatin inside micronuclei. In some micronuclei we found membranes inside the chromatin territory (Fig. 6b - note the membranes inside micronucleus, D - arrowheads), which suggested an invasion of endoplasmic reticulum membranes and might represent nuclear envelope collapse and disruption of micronuclei.

**Figure 7:**
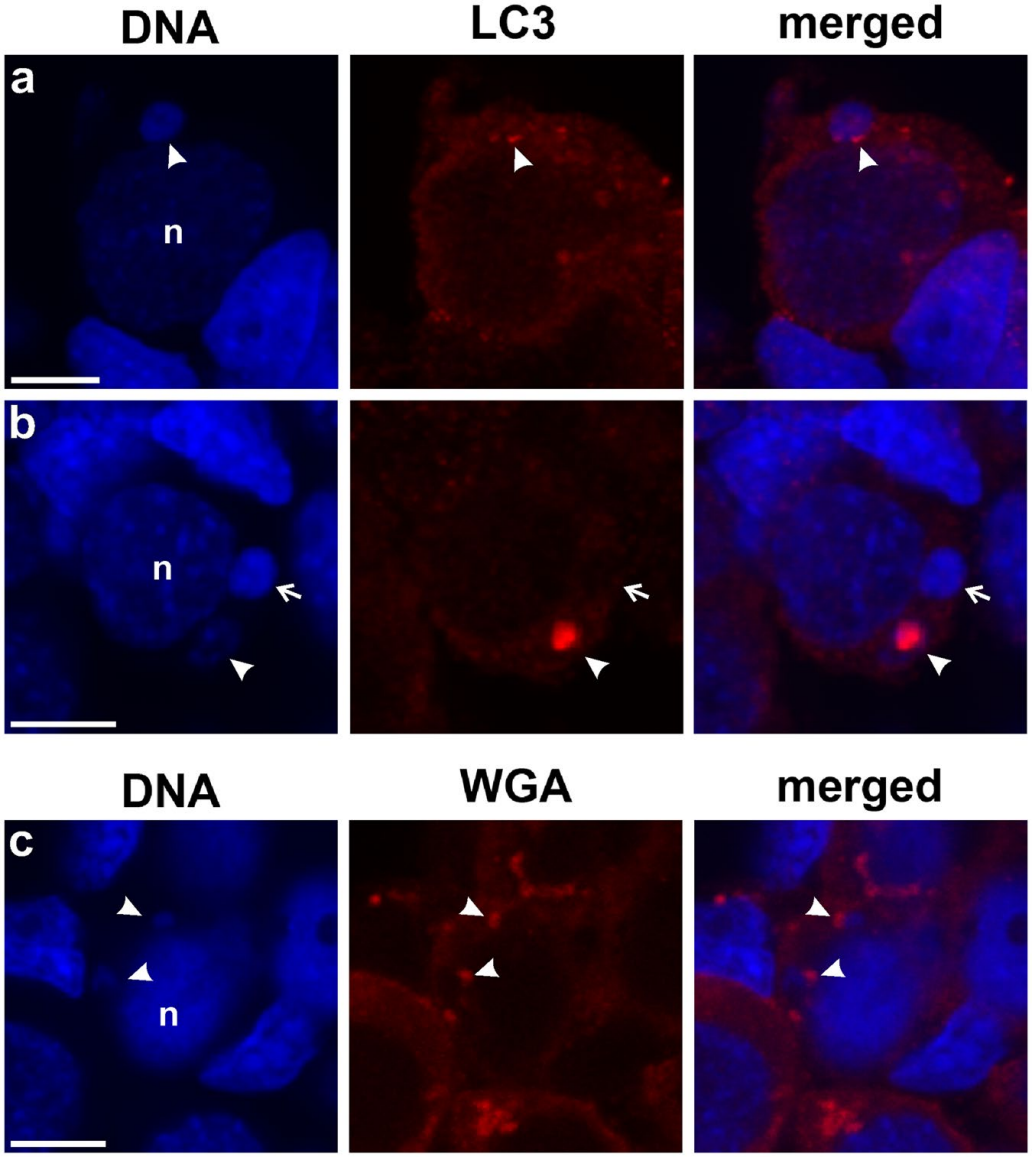
Autophagosome markers are accumulated in the micronuclei. Immunofluorescence staining of paraffin sections from gonads of diploid female at 35 Gosner stage: LC3 autophagosome marker (red), DNA counterstained with DAPI (blue) (**a**,**b**). (**a**) Dotted and linear aggregates of LC3 (arrowhead) on the surface of highly condensed micronuclear chromatin may represent the early stage of autophagosome formation. (**b**) Large LC3 aggregate (arrowhead) on the micronucleus and between the chromatin clumps. Note that DNA is less condensed and fragmented with an empty interior which may represent the later stage of autophagic process connected with DNA degradation. Second micronucleus (arrow) is highly heterochromatinized and apparently not autophagic. (**c**) Immunofluorescence of frozen tissue sections of an ovary from diploid female at 29 Gosner stage showing wheat germ agglutinin (WGA, red) staining of cellular membranes, DNA counterstained with DAPI (blue). Note the cap-shaped WGA aggregates (arrowheads) at the surface of micronuclei which we interpret as staining of fusion site of autophagosome membranes. The irregular shape of the left micronucleus could reflect chromatin degradation. Additional strong intra-cytoplasmic agglomerates or dots, visible in upper and lower cells, could be *trans*-Golgi cisternae and post-Golgi membranes, e.g. lysosomal membranes. Confocal images represent projection of 9 z-sections (**a**) or single z-section (**b**,**c**) (0.46 µm). N – nucleus. Scale bars 5 µm.

**Figure 8:**
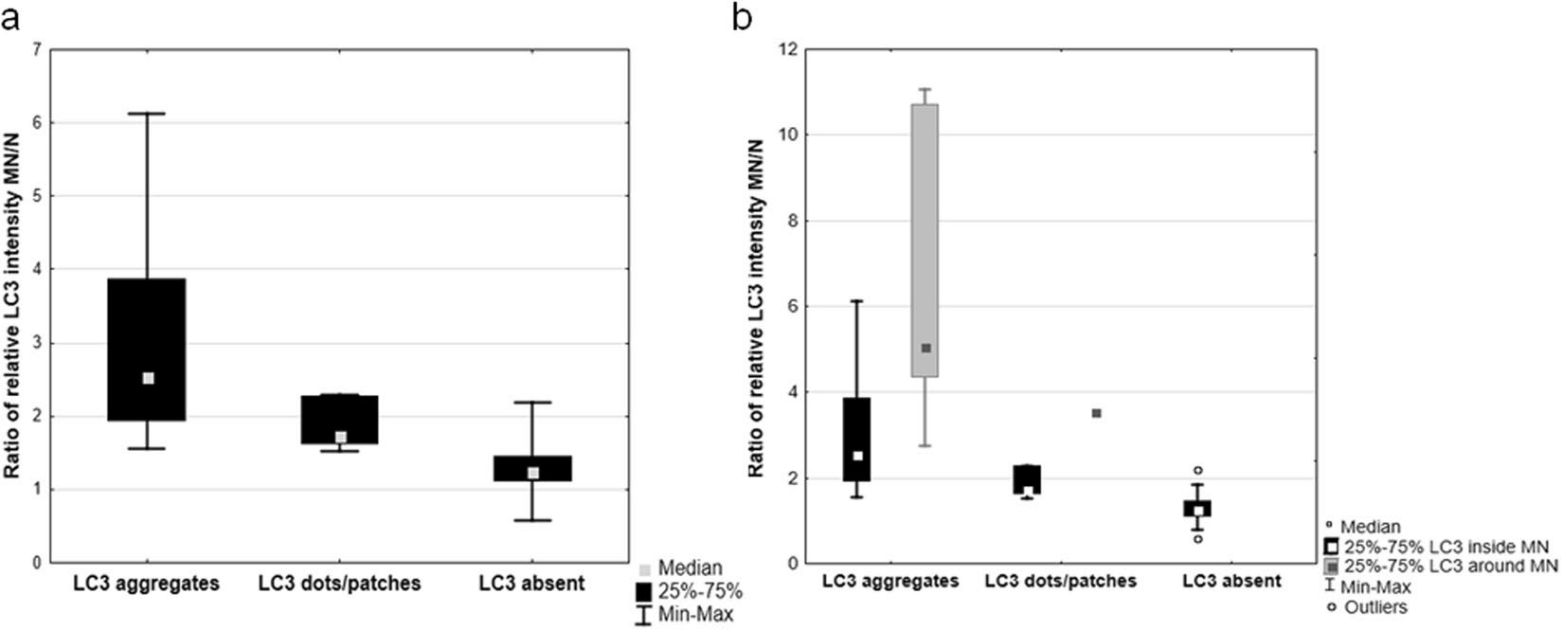
Quantitative analysis of LC3 autophagosome marker presence in micronuclei. (**a**) Ratios of LC3 relative fluorescence of micronuclei (MN) to main nuclei (N) according to 3 classes of LC3 distribution (aggregates, dots/patches, absent) in gonocytes of hybrid frogs. (**b**) Ratios of LC3 relative fluorescence of micronuclei (MN) to main nuclei (N) in gonocytes of hybrid frogs. In case of LC3 aggregates and dots/patches the signal was measured inside whole area of a micronucleus (black bars) or juxtaposed outside the perimeter (solid grey) of chromatin in MN, related to aggregating membranes of forming autophagosomes.

To support the ultrastructure investigation, we applied immunofluorescence examination using microtubule-associated protein light chain 3 (LC3) as the marker of phagophore and autophagosome formation. The cytoplasm of gonocytes in hybrid gonads (Fig. 7a,b), as well as in *P. lessonae* and *P. ridibundus*, was filled with punctate signals, corresponding to the free form of LC3 proteins residing in that compartment. The prevailing portion of micronuclei (76%) displayed no LC3 signal whereas 14% had only weak punctate signals, similar to the free cytoplasmic form of the protein. Nevertheless, in 10% of all observed micronuclei (9 of 68 cells analysed), chromatin was decorated by LC3 aggregates corresponding to the membrane-bound form of LC3. The aggregates were visible as strong dots situated at the periphery of highly condensed chromatin in micronuclei (Fig. 7a - arrowhead) or as clumps at one of their sides; at the same time the lower density and fragmentation of chromatin inside the micronuclei were observed (Fig. 7b - arrowhead). This may represent stages of phagophore and nucleophagosome formation, respectively, as well as chromatin degradation inside micronuclei. To confirm the presence of intracellular membranes surrounding micronuclei we applied lectin cytochemistry using wheat germ agglutinin (WGA), a useful marker of plasma membranes, as well as post-Golgi membranes and phagophore structures in cytoplasmic compartment of a cell. In some cases we found micronuclei accompanied by a patchy WGA signal at one of their sides; which may represent the Golgi-derived membranes of autophagosome forming around micronuclei (Fig. 7c - arrowheads). We supported morphological observations by quantitative densitometric analysis of relative fluorescence signal of LC3 present inside nuclei, micronuclei, and around micronuclei, i.e. in the area of intensive signal reflecting the nucleophagophore membranes (ratio of mean fluorescence values of micronucleus to nucleus (see Supplementary Table S4). The ratios were significantly different in the three analysed groups of micronuclei, the highest values were found in micronuclei possessing LC3 aggregates, lower values were noted in micronuclei displaying dots and patches, and the lowest values in micronuclei without nuclear envelope signal (see Fig. 8a). Within the group of LC3 aggregates we also measured the signal intensity around the micronuclei, which was about twice as high as inside micronuclei (Fig. 8b). We performed TUNEL assay on paraffin sections of gonads followed by LC3 immunocytochemistry to answer the question whether chromatin in micronuclei is experiencing degradation. However, our results shown in Supplementary Fig. S7 and Supplementary Table S5 did not reveal TUNEL signal in micronuclei, while apoptotic gonocytes showed accumulation of double strand breaks in nuclear DNA. Similarly we could not detect LC3 signals in micronuclei when a staining was applied subsequently after TUNEL assay. However, we cannot exclude that Proteinase K digestion of the tissue during TUNEL protocol affected LC3 molecules, which are small proteins, and potentially may have lost their affinity to antibodies. Taken together, these results show possible nuclear envelope collapse and support our hypothesis of the autophagic removal of micronuclei, since DNA-nicks generated by the lysosomal enzyme deoxyribonuclease II did not allowed for TUNEL detection.

### Micronucleated gonocytes and apoptosis

**Figure 9:**
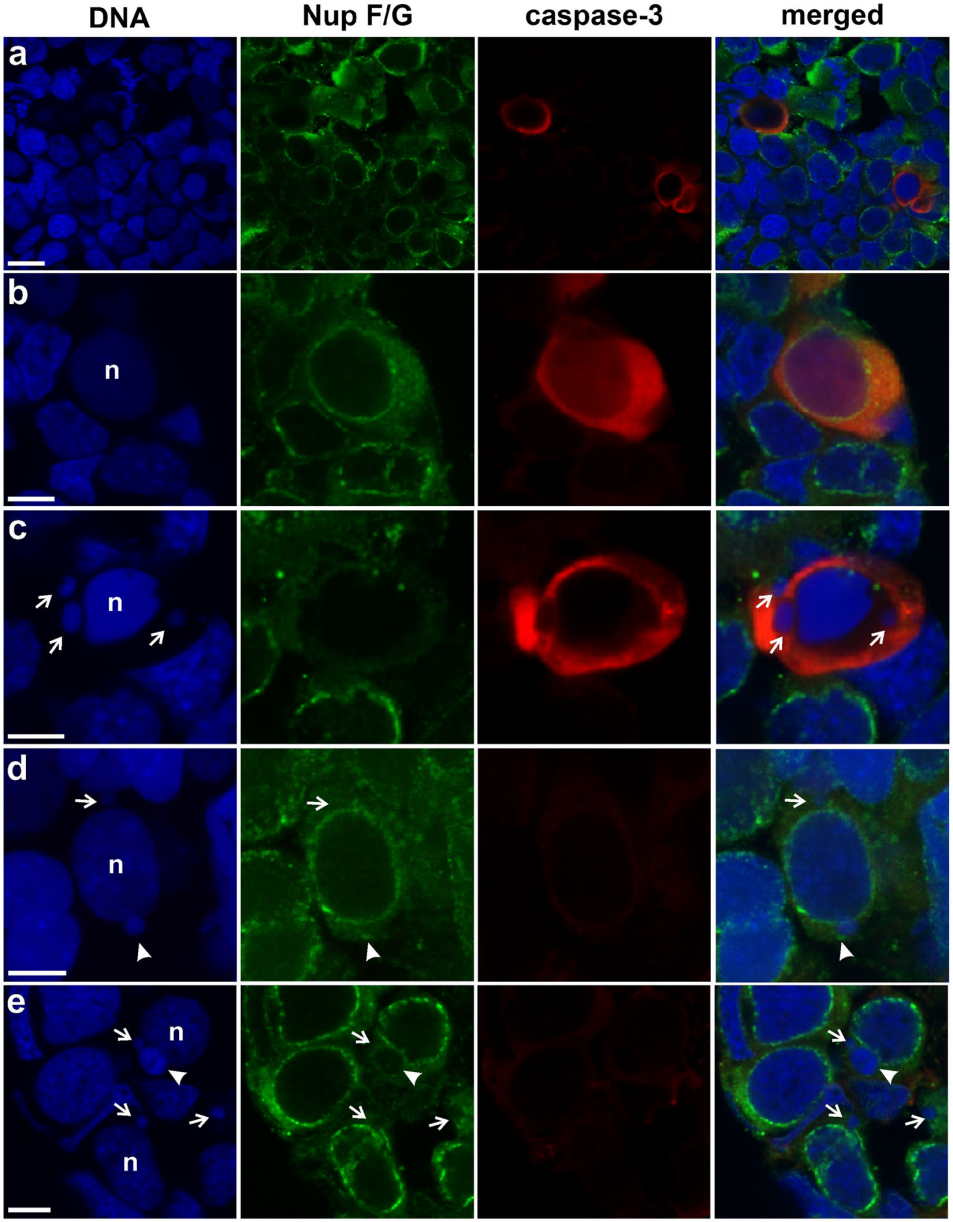
Presence of micronuclei does not lead to apoptotic death of gonocytes. Immunofluorescent staining of frozen tissue sections of gonads from *P. esculentus* diploid female (**a**–**c**, **e**) and male (**d**) at 34 Gosner stage: active caspase-3 (red), NPC proteins (Nup F/G, green), DNA counterstained with DAPI (blue). Gonocytes with positive expression of active caspase-3 showing various stages of degeneration: (**a**) the view of ovary section with interphase, mitotic and apoptotic germ cells, (**b**) germ cells possessing NPC proteins at the NE of nucleus containing flat/amorphous chromatin without heterochromatin/euchromatin structure and similar DNA condensation level as surrounding germ cells, may reflect the early stage of apoptosis (**c**) germ cell with pycnotic nucleus and three micronuclei completely devoid of NPC proteins (arrows) showing later stage of apoptosis. Apoptotic cells possessing micronuclei were very rare in gonads. (**d,e**) Majority of germ cells possessing micronuclei of various sizes and NPC proteins level represent the normal cellular morphology and absence of active caspase-3 apoptotic marker, suggesting that DNA elimination in micronuclei does not affect normal cellular processes. (**d**) Micronucleus with condensed chromatin decorated by NPC proteins dots (arrowhead), second micronucleus is lacking NPC proteins, (**e**) big micronucleus with highly condensed chromatin and weak NPC proteins signal at the nuclear membrane (arrowhead) and small micronuclei lacking NPC proteins signal (arrows). Confocal images represent single z-section (**a–c,e**) or projection of 12 z-sections (**d**) (0.46 µm) to image micronuclei at different levels in the cell. Scale bars (**a**) 10 µm, (**b**–**e**) 5 µm.

To find out whether micronuclei formation was a possible cause of gonocyte death, we analysed the indirect immunofluorescence labelling of cells with antibodies to active caspase-3, which is a reliable marker of apoptosis. Cells were assessed as apoptotic when active caspase-3 staining was present in the cytoplasm and chromatin structure in cell nuclei was amorphous and condensed, and thereby lacking heterochromatin/euchromatin structure, as compared to surrounding germ cells. In the parental species gonads, where micronuclei were absent, we did not found apoptotic signals in the gonocytes (Supplementary Fig. S3); the only exceptions were gonocytes situated in the degenerating distal portion of the testes, a normal process of male gonad morphogenesis (*P. lessonae*, Supplementary Fig. S3; see^61,64^). On the contrary, we found numerous apoptotic gonocytes in hybrid gonads (Fig. 9a–c, see also the recent publication of our team^65^). Among this group there were cells possessing FG-repeat nucleoporins at the nuclear membrane of nucleus (Fig. 9b), or lacking the nuclear rim staining (Fig. 9c). We found only two gonocytes displaying strong active caspase-3 signal in the cytoplasm, apoptotic morphology, and the presence of 2 or 3 spherical chromatin blebs, 0.6–1 µm in diameter, which might be considered as micronuclei (Fig. 9c).

We analysed 31 micronucleated cells that did not exhibit active caspase-3 signal. Morphology and nuclear structure was normal and similar to surrounding gonocytes. We could detect strong expression of FG-repeat nucleoporins at the nuclear envelope of the main nucleus. Among this group of cells, the nuclear envelope in micronuclei had reduced level or punctate signals of NPC proteins (Fig. 9d,e - arrowhead), but with similar frequency we could also find micronuclei without FG-repeat nucleoporins (Fig. 9d,e - arrows). Despite of chromatin degeneration and reduced FG-repeat nucleoporin expression in micronuclei, no active caspase-3 signal was present in the cells. These results demonstrate that micronuclei formation as such was not related to apoptosis and thereby should be considered as a unique physiological process characteristic of *P. esculentus* hybrid gametogenesis.

## Discussion

Micronuclei have never been observed in the parental species, and have never been reported in gonocytes of any amphibian species, neither in primary oogonia nor in prespermatogonia. In the ranid frogs, they apparently are not nuclear lobes because the shape of gonocytes’ nuclei is mostly spherical or ovoid, and only slightly irregular in outline, but not lobed as is the case of most amphibian species^56–58,61^. The same was confirmed in the present paper using confocal microscopy of wheat germ agglutinin stained gonads where all encountered micronuclei were situated between the nucleus and the cell membrane. Ultrastructural examination of the hybrid gonocytes revealed that the nuclear membrane, at least the lipid bilayer, was well preserved in the majority of micronuclei, but the nuclear pore complexes were absent or observed occasionally. The reduced density and abnormal distribution of pore complex structures in the micronuclear envelope was further confirmed in immunofluorescence approach by the use of FG-repeat nucleoporins as a nuclear membrane marker^66,67^. Unfortunately, widely used lamin B1 antibodies were not useful in gonocytes, although they gave strong signal in somatic cells (data not shown). The reduced pore density was found in the radiation-induced micronuclei in mammalian cells^68^, moreover in the sea lamprey, micronuclei did not possess neither lamin B1 nor nuclear pore O-linked glycoproteins^52^. These alterations of nuclear envelope were connected with subsequent chromatin degradation in micronuclei as was also shown by other authors for lamin B1^69^. These findings support our point of view that disruption of nuclear pore complex structures may precede chromatin degradation in micronuclei in hybrid gonocytes.

Heterochromatinization of genetic material destined for elimination accompanies almost all known cases of programmed DNA elimination in different organisms^52,70–72^. During elimination of germline-restricted chromosomes in the finches and the *Sciara* flies, and whole paternal genome elimination in mealy bugs *Planococcus citri*, strong heterochromatinization and abnormal H3S10 phosphorylation caused improper chromatin compaction before mitosis or meiosis. As a result, chromosomes failed to attach to the spindle microtubules and thereby were lost^5,70,73–75^. During the programmed genome rearrangement in the sea lamprey DNA accumulation of H3K9me3 and 5MeC heterochromatinization marks in micronuclei might play functional roles in maintaining chromatin compaction or positioning the eliminated chromatin^52^. It seems evident that formation of micronuclei in gonocytes of the hybridogenetic *P. esculentus* follows the same cellular mechanisms as in other organisms, as well as in cancer cell lines, i.e. during both the programmed and non-programmed DNA elimination. The lower level or absence of H4Ac and accumulation of H3K9me3 histone methylation confirmed transcriptional silencing and heterochromatinization of chromatin encapsulated in micronuclei. Our data from TEM showed both eu- and heterochromatic micronuclei content, which suggests that micronuclei are first detached and then heterochromatinized. However, at the moment we cannot unambiguously state when heterochromatinization of the eliminated DNA starts. Together with different levels of heterochromatinization observed ultrastructurally and by means of immunofluorescence densitometric analyses, we can assume that micronuclei contain chromatin that no longer plays its function and is directed for degradation.

In the present study we confirmed our initial suggestion about formation of micronuclei in gonocytes during interphase via budding from the main nucleus^34,54^. We found several gonocytes with micronuclei budding from the main nucleus, where chromatin was in tight contact with the nuclear chromatin mass (for details see Fig. 5c–f). The nuclear envelope surrounding chromatin at the budding site was depleted of majority of nuclear pore complex proteins, which might explain the low level of pore complexes observed in the prevailing group of micronuclei. Even more severe disruption of micronuclear membrane lacking lamin B1 and nuclear pore O-linked glycoprotein was observed in the sea lamprey somatic cells where micronuclei were formed from lagging chromatin during mitosis and were encapsulated *de novo* by nuclear membranes^52^. At the earliest stages of ovaries and testes differentiation studied herein we did not find lagging chromosomes in the gonocytes of diploid and triploid hybrids; the same observations were provided by Dedukh *et al*.^55^. However, lagging chromosomes were observed in some gonocytes at later stages around completion of metamorphosis^34,54^, so we cannot rule out the possibility of mitotic micronuclei formation. Moreover, they do not stand in contradiction with each other: budding from interphase nuclei is the primary event, whereas the post-mitotic encapsulation of chromosomes lagging behind the mitotic spindle is the secondary event. Coexistence of the two processes of micronuclei formation was previously reported in interspecific hybrids of plants^49^. Most probably, the elimination of chromatin in hybridogenetic frog gonocytes is a multistep process, which is not always precise and some chromosomes may lack active centromeres; to solve the problem, a more detailed study focused on mitotic spindle organization, centromere distribution, and lagging chromosomes is needed.

**Figure 10:**
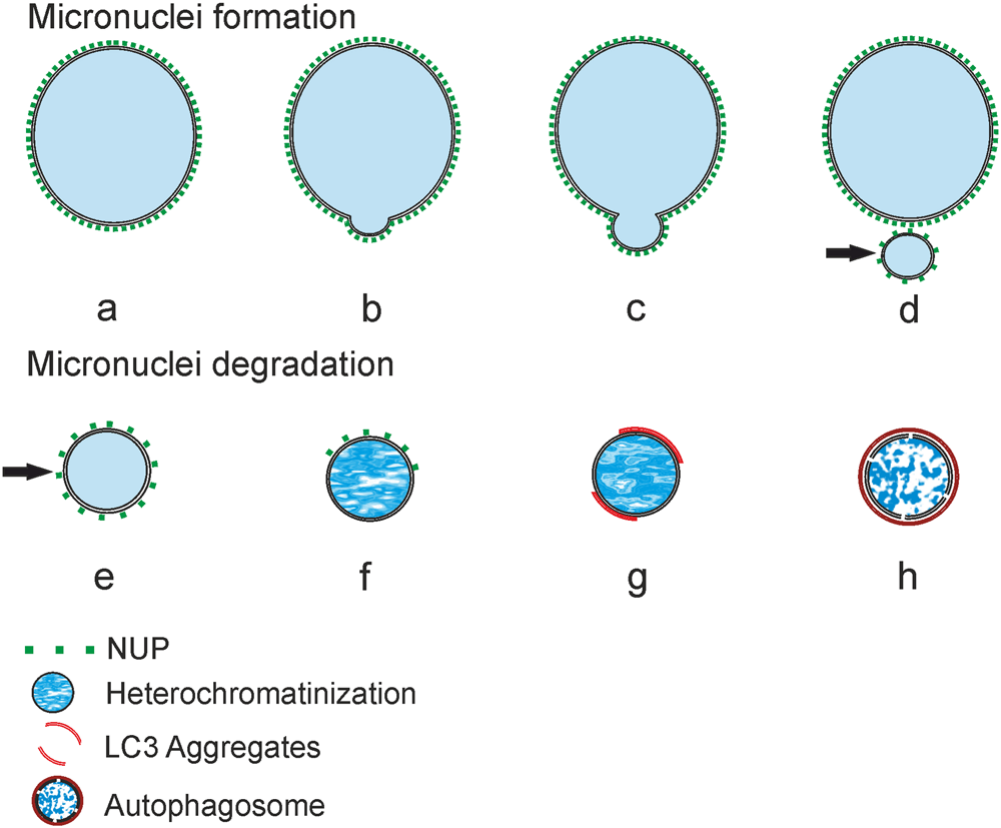
(**a**) diagram summarizing the supposed process of micronuclei formation (**a**–**d**) and degradation (**e**–**h**) in gonocytes of a hybrid *P. esculentus*. (**a**) Gonocyte nucleus with NPC proteins in nuclear envelope (green), (**b,c**) emerging chromatin extrusions (buds), (**d,e**) fully separated micronucleus with lower expression of NPC proteins, (**f**) heterochromatinized micronucleus possessing partial NPC signal, (**g**) strongly heterochromatinized micronucleus with LC3-positive membranes (phagophore, red), (**h**) nucleophagic micronucleus with abnormal chromatin structure and disrupted membranes.

Rello-Varona *et al*.^76^ reported that discontinuity of the nuclear membrane, reduced density of chromatin, and presence of lysosomal vesicles engulfed in autophagosomes around micronuclei indicated their degradation. We obtained similar results and confirmed that micronuclei in the hybrid gonocytes are directed to autophagy. The TEM images of gonocytes revealed considerable amount of membranes lying around micronuclei that were most probably precursors of autophagosomes. We found structures representing all steps of autophagy^63,77,78^ beginning from cytoplasm sequestration to phagophore, and finally to autophagosome formation. Autophagic degradation of micronuclei was also confirmed by LC3 immunostaining. This protein is present in the cytoplasm in a free form and is aggregating on the membranes during formation of phagophore and further autophagosome closure^53,79^. In our study only 10% of all micronuclei displayed LC3-positive aggregates, which may reflect a rapid turnover of autophagic micronuclei, as suggested by Rello-Varona *et al*.^76^. Autophagy is also playing an important role in the programmed DNA elimination in a unicellular eukaryote *Tetrahymena thermophila*, in which several nuclei are selectively degraded during sexual reproduction^80,81^. The next evidence for autophagic removal of micronuclei in hybrid frogs comes from lectin cytochemistry. WGA-positive vesicles accompanying micronuclei may represent either staining of the terminal portion of the isolation membranes (phagophore) assembling on the surface of micronuclei, or the fusion site at the rims of closing nucleophagosome membranes. These results are consistent with TEM images showing the lipid double membranes surrounding some micronuclei. Similar staining pattern was obtained in cardiomyocytes^82^, and in Golgi vesicles and autophagosomes of rat hepatocytes^83^, where autophagic degradation was confirmed. Except structures typical of autophagy, we also noticed membranes invading chromatin inside some micronuclei. Invasion of membranes may indicate abnormal nuclear lamina organization and subsequent chromatin degradation^69^. Moreover, invagination of nuclear membrane was also reported during micronuclei formation via budding of B chromosome from interphase nuclei in *Sciara coprofila*^51^. Chromatin encapsulated in micronuclei from *P. esculentus* gonocytes does not accumulate double strand breaks (no TUNEL reaction) characteristic of apoptosis and hydrolysis by deoxyribonuclease I (DN-ase I), the enzyme generating 3′-hydroxyl groups during DNA fragmentation. Our data are in contradiction with the majority of reports where micronuclei formed from lagging chromosomes or budding from interphase nuclei display TUNEL signal^49,52,84^. The study of Hatch *et al*.^69^ does not report DN-ase I cleavage of chromatin in micronuclei during catastrophic nuclear envelope collapse. The evident lack of this cleavage in the case of micronuclei in hybrid frogs can indirectly suggest DNA degradation by autophagy, where lysosomal acidic hydrolase, deoxyribonuclease II (DN-ase II), is active, producing 3′-phosphate groups in digested DNA molecules which are not detected in TUNEL assay^85,86^. This evidence implicates that micronuclei are more likely degraded via autophagy and not by apoptotic-like processes. The final fate of micronucleated chromatin needs further investigation in terms of other autophagy markers (Beclin-1 and 2, Atg proteins family) and presence of lysosomal membrane markers (LAMP2), as well as accumulation of γH2AX as a marker of DNA repair. Summarizing our observations of nuclear pore complex depletion and degradation of micronuclei, we can present the following scenario of micronuclei fate: from budding and detachment of chromatin during interphase to final degradation by nucleophagy (as summarized in Fig. 10).

A recent study on cancer cell lines experimentally induced to form micronuclei by drug treatment reported the coincidence of apoptosis and the presence of micronuclei^87^. The authors favoured explanation that the malfunctioning of gene expression in the micronuclei might trigger apoptosis, but they could not exclude the harmful effect of severe DNA damage in micronuclei. In the case of hybrid frog early gametogenesis we proved that the prevailing group of micronucleated gonocytes was not directed to apoptosis, so we assume that micronuclei formation during hybridogenetic frog gametogenesis is a physiological process. Degenerating organelles are neutralized and eventually the activation of cell death is prevented due to autophagy that plays a role of the survival factor^63,88^. Because the majority of gonocytes survives at least during premetamorphosis and early gonad development, we suppose that micronuclei formation is a part of the programmed genome rearrangement. The presence and further disruption of micronuclei do not necessarily lead to cell death and do not prevent cell divisions^47,52,69^ as also shown in our study. In hybridogenetic frogs, it seems probable that gonocytes in developing gonads would divide several times until the whole DNA of the eliminated genome is removed. Micronuclei were detected at similar time of tadpole development (around metamorphosis) when Tunner and Heppich^35^, Heppich *et al*.^89^, and Tunner and Heppich-Tunner^36^ reported the gradual loss of the *lessonae* chromosomes from the gonocytes of *P. esculentus*. The process of genome elimination in water frogs seems to be long and inaccurate, although the production of micronuclei as such do not cause gonocyte death. Development and differentiation of gonads in both sexes are delayed at least one year, as compared to the parental species, and a great bulk of germ cells degenerate at this time^34,65,90^. This raises the question, which cellular mechanisms may be compromised during the genome elimination. We can only hypothesize that the loss of some genes maintaining the proper cell cycle progression or required for cell viability, which were entrapped into micronuclei, exposes possible deleterious mutations on the clonally transmitted chromosomes and eventually leads to apoptotic cell death^87,91^.

Micronuclei, putatively carrying the eliminated genome, have never been observed in somatic cells of hybridogenetic animals although as a rule they have two different chromosome sets: one from the mother and the second from the father. The homeologue chromosomes of both the parental species of *P. esculentus* are similar, however, in the *ridibundus* chromosomes heterochromatin is more abundant, DNA content is higher, and they are longer than the *lessonae* ones^23,92–95^. Apparently, the chromosome incompatibility as such is not the only prerequisite for genome elimination from the germline cells. We suspect that there is an unknown mechanism(s) that acts only in cells committed to undergo meiosis that may be triggered during gonocyte proliferation.

## Conclusions

Chromatin elimination via micronuclei in *P. esculentus* is unique among hybridogenetic animals and contributes to broadening the knowledge about reproductive modes in animals. Here we show for the first time that formation of micronuclei in early pre-meiotic germ cells (gonocytes) of a hybrid frog is a physiological process that may be considered as the programmed DNA rearrangement leading to eventual elimination. Micronuclei contain chromatin directed for degradation, which is the essence of genome elimination in hybridogenetic gametogenesis. In the light of our observations presented in this study we suggest the following mechanism of micronuclei formation and degradation in hybrid water frogs: (a) budding of chromatin into micronuclei encapsulated by double membrane with lower density of nuclear pore complexes, (b) subsequent heterochromatinization and loss of transcriptional activity followed by (c) probable degradation of DNA by autophagy (nucleophagy). The process is the same in females and males, both diploid (RL) and triploid (RRL and LLR). Our study on cellular level is a necessary basis for further genetic and epigenetic research on hybridogenesis in *Pelophylax* water frogs. Comparing the mechanism of micronuclei formation, chromatin silencing and heterochromatinization, as well as degradation via autophagy described in hybridogenetic frogs, other organisms, and cancer cells, we conclude that some aspects of the DNA elimination process may be evolutionarily conserved.

## Materials and Methods

### Animals

All procedures with both adults and tadpoles were performed in accordance with relevant guidelines and regulations. The capture of adult frogs was approved by the Polish General and Regional Directorates for Environmental Protection (DOPg 4201-02-74/05, DOP-oz.6401.02.2.2013.JRO, DOP-oz.6401.02.2.2013.2014.JRO.as, WPN.6205.28.2014.IW.2, DZP-WG.6401.02.5.2015.JRO, WPN.6401.177.2016.IL). The adult individuals used for *in vitro* crosses were sampled from different locations in Poland, according to data collected in Supplementary Table S6A. *P. lessonae* (females N = 8, males N = 16) were obtained from populations of L-E and R-E-L type systems: *P. ridibundus* (females N = 11, males N = 6) were collected from R-E and R-E-L population systems. *P. esculentus* (RL females N = 7, RL males N = 3, RLL males N = 1) were taken from E-E population systems in Wysoka Kamieńska from north-western Poland^96^, and Uciechów from south-western Poland, as well as from other locations (Supplementary Table S6B).

All further experimental procedures were accepted by the Local Commission for Ethics in Experiments on Animals in Wrocław, Poland (103/2007, 7/2013, 27/2016). The collected animals were kept in humid boxes and subsequently used for *in vitro* crossing experiments according to the standard procedures for water frogs^97^. Gravid females were injected intraperitoneally with 6,25 mg/kg of body weight salmon luteinizing hormone-releasing hormone (LHRH, H-7525.0001, Bachem) in amphibian PBS (APBS, pH 7.4, 11.2 mM NaCl, 0.22 mM KCl, 0.8 mM Na_2_HPO_4_, 0.14 mM KH_2_PO_4_) 24 hours before the procedure. Males and females were sacrificed after anesthetizing in 0.5% solution of ethyl 3-aminobenzoate methanesulfonate (MS-222, Sigma Chemical Co.) in APBS. Tissues and organs were dissected for DNA (phalange) and chromosomal (intestine) analyses, as well as for *in vitro* fertilization of eggs (testes). Tadpoles of various phenotypes (LL, RR, RL, RRL, RLL), resulting from controlled *in vitro* crosses, were reared in a greenhouse in PPE tanks, in a concentration of c.a. 10 tadpoles per litre of water, and fed with frozen lettuce and fish food. Altogether, 7 tadpoles of *P. lessonae* (progeny of 4 pairs), 2 of *P. ridibundus* (progeny of 2 pairs), and 46 diploid and 11 triploid tadpoles of *P. esculentus* (progeny of 24 pairs) were used (for details see Supplementary Table S7). Staging of somatic development of tadpoles followed Gosner^62^. Gonads for morphological and immunohistochemical analyses were dissected from tadpoles after anesthetizing by immersion in 0.25% solution of MS-222 (Sigma Chemical Co.) in APBS.

### Taxonomic evaluation of animals

Taxonomic evaluation of adult frogs collected for crossing experiments was performed first according to morphological criteria. Ploidy of *P. esculentus* individuals was estimated before performing crosses using erythrocyte long axis measurements according to Kierzkowski *et al*.^98^. The blood smears were obtained after gentle cutting one of the phalanges of a hind limb and air-dried. Measurement of the erythrocytes long axis was performed under Axiostar Plus microscope (Zeiss) using 20× lens and KS400 software (Zeiss). Diploid erythrocytes were 23.4–24.9 µm long and triploid were 29.5–33.3 µm. Species level of parental individuals and tadpoles (used in the examination of nucleoporins, caspase-3 and autophagy) were confirmed by allele size polymorphism in serum albumin introne-1. DNA amplification was done according to Hauswaldt *et al*.^99^ with some modification in PCR protocol following Kolenda *et al*.^100^ (see Supplementary Table S6C). The PCR reaction was performed in a C1000 Thermal Cycler (Bio-Rad, USA): initial denaturation for 3 min at 95 °C followed by 35 cycles of 30 s denaturation at 94 °C, 30 s annealing at 53 °C, and 60 s elongation at 72 °C, and final extension for 7 min at 72 °C. Three µl of PCR products were separated electrophoretically on a 1.5% agarose gel and compared with a 100 bp size marker.

Further analysis of ploidy and genomic composition was done for adult individuals of *P. esculentus* and for progeny sourcing from *P. esculentus* parents, using microsatellites analysis of sequences differentiating ploidy, FISH with telomeric probe or AMD-DAPI (details in Supplementary Tables S6C and S7).

### Microsatellites analysis

Six nuclear microsatellite loci were used: Re1Caga10, RICa1b6, RICa1b5, Ga1a19red, Rid059A, Rrid013A (details in Supplementary Table S6C). DNA amplification was performed in one multiplex PCR set with forward primers fluorescently labelled with 6-FAM, NED, PET and VIC. Reaction was carried out in 6 µl, containing 1 µl DNA, 3 µl 2 × Multiplex PCR Master MIX (EURx Ltd., Poland), 0.06 µl (10 µM) of both forward and reverse primers (a total of 0.72 µl of primers mix). The PCR reaction was conducted in a C1000 Thermal Cycler (Bio-Rad, USA) under following conditions: initial denaturation for 15 min at 95 °C followed by 30 cycles of 30 s denaturation at 94 °C, 90 s annealing at 60 °C, and 60 s elongation at 72 °C, and final extension for 30 min at 60 °C. Separation of PCR products was outsourced at a commercial facility (Genomed, Warsaw, Poland). Ploidy level and genomic composition were determined using GENEMAPPER v. 3.5 (Applied Biosystems, USA).

### Fluorescence *in situ* hybridization

For analysis of epigenetic chromatin modification during early gonadal development, we took *P. lessonae*, as well as diploid and triploid hybrid tadpoles which were identified using FISH with TTAGGG probe. This method is based on difference between parental species in amount and localization of interstitial telomeric sites on NOR-bearing chromosome. Earlier we found that *P. ridibundus* NOR bearing chromosome has two interstitial telomeric sites while *P. lessonae* NOR bearing chromosome - only one interstitial telomeric site. Thus distinguishing of *P. lessonae* and/or *P. ridibundus* NOR bearing chromosomes allows to infer karyotype composition of parental species as well as diploid and triploid hybrid frogs^32,93^.

FISH with single-stranded oligonucleotide telomeric probes (TTAGGG)_5_ conjugated with Cy3 was performed on metaphase chromosomes as described previously^32,93^. Before hybridization metaphase plates were pre-treated with RNase A (100–200 μg/ml) for 1 h, pepsin (0.01% in 0.01 N HCl) for 10 min and then post-fixed in formaldehyde (1% in PBS, 50 mM MgCl_2_) for 10 min. Hybridization mixture (40% formamide, 2.4 × SSC, and 12% dextran sulphate, 5 ng/μl labelled probe and 10–50-fold excess of tRNA) was added to metaphase chromosomes. After denaturation at 82 °C for 5 min slides were incubated at room temperature for 12–18 h. Then slides were washed three times in 2 × SSC at 42 °C. After FISH chromosomal preparations were mounted in DABCO antifade solution containing 1 mg/ml DAPI.

### AMD-DAPI chromosomal staining

The method is based on difference in centromeric regions staining pattern between parental species, where *ridibundus* chromosomes display intensive signals, whereas *lessonae* do not stain or stain weakly. Genome composition was determined on 10–20 metaphase plates. Intestinal epithelial cells were put on a slide in a drop of 70% acetic acid and squashed under a coverslip. The slides were put on dry ice block until frozen, and then coverslips were removed. Chromosomes were stained with Actinomycin D- 4′,6-diamidino-2-phenylindole (DAPI) as described by Heppich *et al*.^89^. Slides were examined using Zeiss Axioskop microscope (Zeiss) equipped with a fluorescence lamp and appropriate filters.

### Transmission electron microscopy

Gonads samples for TEM examination were fixed in 2.5% glutaraldehyde (Serva Electrophoresis, Heidelberg, Germany). After 24 hours, the specimens were washed in the cacodylate or phosphate buffers (0.1 M, pH 7.4, Serva) and postfixed for 1 h in 1% osmium tetroxide (Serva). Subsequently, the specimens were dehydrated in ethyl alcohol and twice in pure acetone (Chempur, Poland). Afterwards samples were embedded in epoxy resin (Epon 812, Serva). Epon blocks were cut into semithin (600 nm thick) sections and stained with toluidine blue (Serva). Finally, ultrathin (50 nm thick) sections were prepared using an ultramicrotomes Power Tome XL (RMC, Tucson, USA) and Reichert Ultracut E. Ultrathin sections were counterstained with uranyl acetate and lead citrate (Serva), and examined in a TEM JEM-1011 (JEOL,Tokyo, Japan) or Zeiss EM 900. Digital micrographs were collected with the use of TEM imaging platform iTEM1233 equipped with the Morada Camera (Olympus, Münster, Germany).

### Immunofluorescence on tissue sections

Immunofluorescence studies were performed on tissue sections of *P. esculentus, P. lessonae* and *P. ridibundus* gonads collected at 28–45 Gosner stages. Nuclear envelope staining was carried out using mouse monoclonal antibodies raised against F/G repeat nucleoporins, mAb414 (diluted 1:250, Abcam ab24609, recommended for *Xenopus leavis*), recognizing proteins of the external cytoplasmic part, central core and inner nuclear basket (Nucleoporins p62, Nup153, Nup214, Nup358), previously used in *Xenopus*^66,67^. Apoptosis was detected using rabbit antibodies recognizing active caspase-3 (diluted 1:250, Abcam ab13847, recommended for *Xenopus*), previously used in *P. esculentus* – complex^65^. A positive control for the proper antibody staining pattern in the gonads were degenerating cells in parental species shortening testes^64^. Autophagy was examined with rabbit antibody anti LC3 (diluted 1:200, Novus Biologicals NB100–2331, predicted to react with *Xenopus* based on 100% sequence homology), recognizing both LC3A and LC3B proteins. Wheat germ agglutinin conjugated with Texas Red (WGA, 5 µg/ml, Molecular Probes/Life Technologies) was used for counterstaining of plasma membrane. WGA can bind sialic acid and N-acetylglucosamine in the integral membrane glycoproteins present in post-Golgi membranes as well as in plasma membrane and is a marker of autophagosome formation^83^.

Gonads were dissected under Stemi Zeiss stereomicroscope from 23 individuals: 3 *P. lessonae*, 2* P. ridibundus* and 18* P. esculentus* (16 RL, 2 RRL). For nucleoporins and caspase-3 staining tissues were fixed in 2% PFA in APBS for 1, 5-2 hours at RT, washed 3 × 15 min in PBS and incubated for 2–4 hours in 12.5% and overnight in 25% sucrose in PBS at 4 °C, then mounted in tissue freezing medium (Leica Biosystems), frozen at −20 °C and stored at −80 °C until analysis. Cryostat sections, 7 µm thick, were cut on CM1850UV microtome (Leica Biosystems), mounted on Superfrost Plus microscope slides (Thermo Scientific), washed in PBS (pH 7.4, 14 mM NaCl, 0.27 mM KCl, 1 mM Na_2_HPO_4_, 0.18 mM KH_2_PO_4_) with 0.05% Tween 20 (Sigma) (PBST), incubated with WGA for 10 min at RT, then permeabilized in 0.5% Triton X-100 (Sigma) in PBS for 15 min at RT. For LC3A/B stainings tissues were fixed for 24 hours in 2% PFA in APBS at 4 °C, washed in 10 changes of PBS, then dehydrated through graded alcohol solutions and xylene and embedded in paraffin. Paraffin tissue sections, 7 µm thick, were cut using RM 2255 microtome (Leica Biosystems), deparaffinized in xylen and hydrated in graded alcohol. Heat induced antigen retrieval was performed in 0.01 M citrate buffer, pH 6.0, at 97 °C for 20 min. After washing with PBST, tissues were blocked in 3% goat serum (Sigma) diluted in PBS for 30 min at RT, then primary antibodies diluted in 1% goat serum in PBST were applied for overnight incubation at 4 °C, followed by PBST washing. Secondary antibodies used were goat anti-mouse Alexa Fluor 488 and goat anti-rabbit Alexa Fluor 546 (Invitrogen, Life Technologies), or donkey anti-rabbit conjugated with CY5 (Jackson Immunoresearch) diluted 1:500–1:2000 in 1% goat serum in PBST containing 0.5 µg/ml DAPI and incubated for 1 h at RT, followed by PBST washes. Sections were mounted in ProLong Gold antifade reagent (Invitrogen, Life Technologies). Gonads were analysed using Olympus FV1000 confocal microscope. 3D models of labelled gonads were generated using Imaris 6.2.1 software (Bitplane).

### 3D immunofluorescent staining (Immunofluorescent staining of whole tissues)

For 3D immunofluorescent staining we used rabbit pАТ ab8898 against H3K9Me3 (Abcam) and mouse pАТ 06-866 against H4Ac (Up-state). 3D immunofluorescent staining was performed on whole gonads dissected from tadpoles of *P. lessonae* (N = 4), diploid (N = 9) and triploid (N = 2) *P. esculentus*. Dissection was carried out under observation on stereomicroscope Leica MZ16. Tissues were fixed in 2% PFA prepared on 1× PBS during 1.5 hours at RT. Tissue fragments were stored in PBS with 0.02% Na azide. Before hybridization tissue fragments were permeabilized in 1% Triton X-100 in PBS for 4–5 hours at RT. Tissues were washed in PBS, blocked in PBS containing 1% blocking reagent (Roche) for 1 hour at RT and incubated with primary antibody (1:200 dilution) overnight at RT. Tissues were washed in PBS, and incubated in corresponding secondary antibodies (Alexa-488-conjugated goat anti-rabbit IgG (Jackson ImmunoResearch Laboratories), Alexa-488-conjugated goat anti-mouse IgG (Molecular Probes) for 8 h at RT. Tissues were washed in PBS, and incubated in PBS containing 1 mg/ml DAPI overnight at RT.

### TUNEL assay

In paraffin sections, apoptosis was assessed by TUNEL technique, using Click-iT Plus TUNEL Assay for *in situ* apoptosis detection with Alexa Fluor 647 dye (Molecular Probes, Life Technologies). The procedure was performed according to manufacturer’s instructions. In four gonads, TUNEL assay was followed by immunofluorescence staining for LC3 according to the procedure described above. Each gonad was evaluated in several sections for presence of apoptotic cells, autophagy and micronuclei in gonocytes. Documentation of results was done using Olympus FV1000 confocal microscope.

### Measurements of micronuclei size and immunofluorescence signal intensity

Measurements of micronuclei diameter and immunofluorescence signal intensity were done in digital confocal images using Fiji software^101^ or LAS software (Leica) (for Nup and LC3 specimens or for histone modifications, respectively). Line tool was used to measure distance between the edges of DAPI signal in micronuclei on z-section showing the maximal diameter and DAPI staining. Results are presented in micrometers.

Intensity of DAPI fluorescence was measured in main nuclei and micronuclei using manual free line drawing tool to mark the region of interest (ROI) within the border of DAPI signal on z-section showing the maximal diameter of the measured structure. Collected data represented the values of relative fluorescence mean grey value from the area selected by ROI. To assess whether micronuclei possessed stronger DAPI signal (reflecting the DNA density in the structure) than main nuclei we calculated the ratio of micronucleus mean fluorescence value to main nucleus mean fluorescence value. Micronuclei were grouped arbitrally within the ranges: up to 0.79 – MN have lower fluorescence than nuclei; 0.8–1.2 – fluorescence levels are similar in MN and nuclei; higher than 1.21 – MN have distinctly stronger DAPI signal than main nuclei.

Nup fluorescence intensity measurements were done in borderline of chromatin area reflecting nuclear envelope Nup signal both in MN and main nuclei. The 2pixel-thick ROI lines were created after transformation of the ROI line set previously for DAPI, and program measured intensities of all pixels in the marked area. Raw data represented the mean grey value, minimum and maximum values of fluorescence intensity in the nuclear envelope area, and they were standardized to the perimeter of the structure by means of FIJI measurements settings. The results are represented as ratio of relative Nup fluorescence of micronuclei to main nuclei. We have compared data sets for groups set previously on basis of qualitative image analysis: (1) Nup staining present in rim area of micronuclei nuclear envelope, (2) Nup staining present as single dots or patches aggregated on one side of MN, (3) no distinct Nup signals detected visually in nuclear envelope.

LC3 fluorescence intensity measurements were done in the same ROI area set initially for DNA measurement in main nuclei and micronuclei. Additionally, LC3 intensity was measured in ROI of LC3 aggregates around micronucleus. The results are presented as ratio of relative LC3 fluorescence of micronuclei to main nuclei. We have compared data sets for groups set previously on basis of qualitative image analysis: (1) strong signal in LC3 aggregates surrounding micronuclei, (2) weak LC3 signals present as single dots or patches aggregated on one side of MN, (3) no distinct LC3 signals around micronuclei.

Estimation of signal intensiveness in the main nuclei and micronuclei after immunofluoresce staining with antibodies against H3K9me3 and H4Ac was made using Leica LasAF software. ROI was selected manually for each micronucleus and corresponding main nuclei. Signal intensity was measured for DAPI channel and channel with fluorescence staining for micronuclei (value IF signal MN; value DAPI signal MN) and main nuclei (value IF signal N; value DAPI signal N). To detect changes in fluorescence intensity for histone marks we used relative staining value of the antibody staining fluorescence (either H3K9me3 or H4Ac) to fluorescence of DAPI (value IF signal MN/value DAPI signal MN; value IF signal N/value DAPI signal N). The comparison of this relative indices between micronuclei and main nuclei allowed to assess fluctuation of the fluorescent staining in the micronuclei comparatively to main nucleus: (value IF signal MN/value DAPI signal MN) – (value IF signal N/value DAPI signal N).

### Statistical analysis

Statistical analysis was performed using STATISTICA 12.5 software (StatSoft, Poland). Groups of data (groups of micronuclei displaying different distributions of NPC and LC3 signal) were compared using the non-parametric Kruskal-Wallis test. Statistical significance was defined when P ≤ 0.05.

### Image processing

Any brightness and contrast adjustments were performed in Adobe Photoshop and Corel Draw.

### Data Availability

All data generated or analysed during this study are included in this published article (and its Supplementary Information files).

## Electronic supplementary material

Supplementary Information

Supplementary Movie 1

Supplementary Movie 2
