# Supplementary material for: The programmed DNA elimination and formation of micronuclei in germ line cells of the natural hybridogenetic water frog Pelophylax esculentus
Source: Sci Rep. 2018 May 18;8:7870. doi: 10.1038/s41598-018-26168-z (PMC5959867; doi:10.1038/s41598-018-26168-z)
Supplement: Supplementary file 1 — Supplementary Information [file 41598_2018_26168_MOESM1_ESM.pdf]

Supplementary Information 1

<u>Title:</u>

The programmed DNA elimination and formation of micronuclei in germ line cells of the natural hybridogenetic water frog *Pelophylax esculentus*

<u>Authors:</u>

Magdalena Chmielewska, Dmitry Dedukh, Katarzyna Haczkiewicz, Beata Rozenblut-Kościsty, Mikołaj Kaźmierczak, Krzysztof Kolenda, Ewa Serwa, Agnieszka Pietras-Lebioda, Alla Krasikova, Maria Ogielska

Supplementary Table S1. Frequency and numbers of various micronuclei sizes in gonocytes of hybrid frogs.

Genome composition	Gosner stage	Number of micronuclei								Total no of analysed MN	No of individuals
		diameter 0.8-1 µm		diameter 1.1-2 µm		diameter 2.1-3 µm		diameter 3.1-4 µm			
		No	%	No	%	No	%	No	%		
RL	28-29	2	4.45	23	51.11	14	31.11	6	13.33	45	2
RL, RRL, RLL 3n	31-33	13	3.53	148	40.22	140	38.04	67	18.21	368	9
RL, RRL, RLL 3n	34-36	16	3.36	160	33.61	188	39.5	112	23.53	476	10
RL	41-46			12	66.67	5	27.78	1	5.55	18	2
**Total**	**28-46**	**31**	**3.42**	**343**	**37.82**	**347**	**48.26**	**186**	**20.5**	**907**	**23**

Supplementary Table S2. Number of micronuclei in gonocytes of hybrid frogs during development according to Gosner stages (G)

Genome composition	Gosner stage	Number of gonocytes with micronuclei										Total no of MN cells		No of individuals
		1 MN/cell		2 MN/cell		3 MN/cell		4 MN/cell		5 MN/cell				
		No	%	No	%	No	%	No	%	No	%	No	%	
RL	28-29	28	62.22	9	20	8	17.78					45	100	3
RL, RRL, RLL 3n	31-33	173	66.54	56	21.54	26	10	5	1.92			260	100	10
RL, RRL, RLL 3n	34-36	238	69.59	72	21.05	26	7.6	6	1.76			342	100	10
RL	41-46	20	55.56	11	30.56	2	5.55	1	2.78	2	5.55	36	100	4
**Total**	**28-46**	**459**	**67.2**	**148**	**21.67**	**62**	**9.08**	**12**	**1.76**	**2**	**0.29**	**683**	**100**	**27**

Supplementary Table S3. Ratios of Nup relative fluorescence of micronuclei (MN) to main nuclei in gonocytes of hybrid frogs

	Micronuclei		Mean	Median	Min	Max	SD
	No	%					
Nup rim	5	10.42	1.124	0.940	0.858	1.518	0.315
Nup dots/ patches	20	41.66	0.64	0.55	0.40	1.09	0.20
Nup absent	23	47.92	0.546	0.547	0.287	0.921	0.18

Supplementary Table S4. Ratios of LC3 relative fluorescence of micronuclei (MN) to main nuclei (N) in gonocytes of hybrid frogs

	Number of micronuclei	Mean	Median	Min	Max	SD
LC3 aggregates	7	3.00	2.54	1.55	6.12	1.56
LC3 aggregates around micronuclei	6	6.51	5.08	2.74	11.1	3.51
LC3 dots/ patches	5	1.89	1.73	1.53	2.30	0.37
LC3 dots/ patches around micronuclei	1	3.53	3.53	3.53	3.53	-
LC3 absent	20	1.29	1.25	0.58	2.19	0.36

Supplementary Table S5. Micronuclei do not accumulate double strand breaks in gonocytes of hybrid frog

TUNEL assay has not shown positive reaction in micronuclei in any of examined individuals of hybrid frogs.

Taxon	Gosner stage	No of cells	No of MN	TUNEL signal in MN	No of individuals
RL	28-29	31	42	0	1
RL, RRL	30-33	69	80	0	3
RL, RLL	34-36	36	40	0	3
RL	37-40	8	12	0	1
**Total analyzed**	**28-40**	**144**	**174**	**0**	**8**

Supplementary Table S6: Parental individuals of water frogs *Pelophylax esculentus* – complex used in crossing experiments. (a) Place of origin and taxonomic evaluation methods used for listed animals: Microsat. - assessement of species using PCR method for PelSAI-1 followed by microsattelite analysis of ploidy, Morphol. – morphological assessement of parents used in crossings, FISH – species and ploidy assessement using FISH with telomeric probe. (b) Geographic coordinates for origin of water frogs. (c) Primer information for markers used for taxonomic evaluation of animals using PCR method for PelSAI-1 and microsattelite analysis of ploidy level and genomic composition.

a)

Taxon	Place of origin	Female no.	Genotyping method	Female genotype	Male no.	Genotyping method	Male genotype
Pelophylax	Kotowice	2					
esculentus		2 RL	morphol	RL			
		5 RL	morphol	RL			
	Ruda Milicka				1		
					72 RL	morphol	RL
	Uciechów	2					
		2/16 RL	FISH	RL			
		12/16 RL	Microsat	RL			
	Wysoka Kamieńska	3			3		
		8/2n/15 Bacz 8	Microsat	RL	28/16 RL	FISH	RL
		5/2n/15 Bacz 8	Microsat	RL	41/16 RL	FISH	RL
		1/ 16 RL	FISH	RL	6/16 RLL	FISH	RL
Pelophylax	Domaszczyn	2			3		
lessonae		27/16 LL	PelSAI-1	LL	13/16 LL	PelSAI-1	LL
		42/16 LL	PelSAI-1	LL	49/16 LL	PelSAI-1	LL
					56/16 LL	PelSAI-1	LL
	Kotowice				2		
					6 LL	morphol	
					8 LL	morphol	
	Poznań Rogaczewo	1			2		
		L2	morphol	LL	L5	morphol	LL
					L6	morphol	LL
	Raków	2			6		
		10/15 LL	PelSAI-1	LL	1/15 LL	PelSAI-1	LL
		11/15 LL	PelSAI-1	LL	2/15 LL	PelSAI-1	LL
					15/15 LL	PelSAI-1	LL
					17/15 LL	PelSAI-1	LL
					68 LL	morphol	LL
					53 LL	morphol	LL
	Sanie	1			2		
		10/16 LL	PelSAI-1	LL	4/16 LL	PelSAI-1	LL
					11/16 LL	PelSAI-1	LL
	Urwitałt	2			1		
		13/14 LL	PelSAI-1	LL	2/14 LL	PelSAI-1	LL
		14/14 LL	PelSAI-1	LL			
Pelopylax	Ruda Milicka	6			4		
ridibundus		1/14 RR	PelSAI-1	RR	16/14 RR	PelSAI-1	RR
		4/15 RR	PelSAI-1	RR	6/15 RR	PelSAI-1	RR
		5/15 RR	PelSAI-1	RR	16/15 RR	PelSAI-1	RR
		72 RR	morphol	RR	21/15 RR	PelSAI-1	RR
		69 RR	morphol	RR			
		52 RR	morphol	RR			
	Raszków	2			1		
		R1	morphol	RR	R1	morphol	RR
		R2	morphol	RR			
	Sanie	3			1		
		38/16 RR	PelSAI-1	RR	39/16 RR	PelSAI-1	RR
		43/16 RR	PelSAI-1	RR			
		51/16 RR	PelSAI-1	RR			

b)

**Locality**	**Population type**	**GPS coordinates**
Domaszczyn	L-E	N: 51°11'32.39" E: 17° 9'37.76"
Kotowice	L-E	N: 51° 2'17.81" E: 17° 9'57.08"
Poznań Rogaczewo	L-E	N: 14°51'48.25" E: 16°42'42.44"
Raków	L-E	N: 51°10'25.90" E: 17°16'38.71"
Raszków	R-E	N: 51°42'38.96" E: 17°43'23.51"
Ruda Milicka	R-E	N: 51°31'59.35" E: 17°20'6.42"
Sanie	R-E-L	N: 51°24'24.26" E: 16°56'33.79"
Uciechów	E-E	N: 50°44'39.72" E: 16°42'6.14"
Urwitałt	L-E	N: 53°48'20.62" E: 21°38'29.22"
Wysoka Kamieńska	E-E	N: 53°49'34.83" E: 14°51'48.25"

c)

**Marker**	**Primer sequences**	**Forward primers labels**	**References**
Serum albumin intron-1	F: TCCATACAAATGTGCTAAGTAGGTT	-	Hauswaldt et al. 2012 [1]
	R: GACGGTAAGGGGACATAATTCA		
Microsatelites			
Re1Caga10	F: CATGTTTACCGTCACTTTAAGAACAC	PET	Arioli 2007[2]
	R: CATCTCTTCAGGTGGCTGGA		
RICA1b6	F: AAACTCGCGGTTTCCCTTA	NED	Arioli 2007[2]
	R: GAGCCAGGTTAAGATAACTGGA		
Ga1a19red	F: GCACACTATTTCTGCTGTATTGC	6-FAM	Arioli 2007[2], Christiansen 2009[3]
	R: CAGGGGATTTTCCCATCAG		
Rid059A	F: TGTACCCGTCATCGCTAGAG	VIC	Hotz et al. 2001[4]
	R: CCCCATACATATTGTTGGTTCC		
Rrid013A	F: CGAGAATCGAAGTGGAGAGG	PET	Hotz et al. 2001[4]
	R: ACCCGTCTCCACAATACTGC		
RICA1b5	F: CCCAGTGACAGTGAGTACCG	NED	Garner et al. 2000[5]
	R: CCCAACTGGAGGACCAAAAG		

Supplementary Table S7: Tadpoles of water frogs *Pelophylax esculentus* – complex used in the study. FFPE – formalin fixed paraffin embeded tissue, Karnov. – tissue fixed with Karnovsky solution and embeded in epon, PFA-fro – PFA fixed frozen tissue, PFA-WhM - PFA fixed whole mount tissue, Morphol. – morphological assessement of parents used in crossings, Microsat. - assessement of species using PCR method for PelSAI-1 followed by microsattelite analysis of ploidy, FISH – species and ploidy assessement using FISH with telomeric probe.

Individual	F parent taxon	origin	M parent taxon	origin	tadpole takson	tadpole genotyping method	Gosner stage	Tadpole sex	gonad fix. method	analytical method
**IF paraffin**										
1052/32b/15	RL	Wysoka Kamieńska	LL	Raków	RLL	PelSAI-1 of parents	34	M	FFPE	TUNEL-LC3
1064/1/15	RR	Ruda Milicka	LL	Raków	RL	PelSAI-1	35	F	FFPE	LC3
1064/1/15	RR	Ruda Milicka	LL	Raków	RL	PelSAI-1 of parents	35	F	FFPE	TUNEL
1073/2/15	RR	Ruda Milicka	LL	Raków	RL	PelSAI-1	33	M	FFPE	LC3
1087/23/15	LL	Raków	LL	Raków	LL	PelSAI-1	32	F	FFPE	LC3
1087/23/15	LL	15/L/15	LL	Raków	LL	PelSAI-1 of parents	32	F	FFPE	TUNEL
1183/35/15	RL	Wysoka Kamieńska	RR	Ruda Milicka	RRL	Microsat.	35	M	FFPE	LC3
1183/35/15	RL	Wysoka Kamieńska	RR	Ruda Milicka	RRL	PelSAI-1 of parents	35	M	FFPE	TUNEL
1289/30b/15	RL	Wysoka Kamieńska	LL	Raków	RLL	PelSAI-1 of parents	40	M	FFPE	TUNEL
432/16/14	LL	Urwitałt	RR	Ruda Milicka	RL	PelSAI-1	36	M	FFPE	LC3
559/26/15	LL	Raków	RR	Ruda Milicka	RL	PelSAI-1	30	F	FFPE	LC3
575/1/14	RR	Ruda Milicka	LL	Urwitałt	RL	PelSAI-1	46	M	FFPE	LC3
675/10/15	RR	Ruda Milicka	RR	Ruda Milicka	RR	PelSAI-1	30	F	FFPE	LC3
761/30a/15	RL	Wysoka Kamieńska	LL	Raków	RL	PelSAI-1 of parents	30	F	FFPE	TUNEL-LC3
770/28/15	LL	Raków	LL	Raków	LL	PelSAI-1	30	F	FFPE	LC3
845/30a/15	RL	Wysoka Kamieńska	LL	Raków	RL	PelSAI-1 of parents	28	M	FFPE	TUNEL-LC3
915/26/15	RR	Ruda Milicka	LL	Raków	RL	PelSAI-1 of parents	33	F?	FFPE	TUNEL-LC3
922/34b/15	RL	Wysoka Kamieńska	RR	Ruda Milicka	RRL	Microsat.	32	F	FFPE	LC3
922/34b/15	RL	Wysoka Kamieńska	RR	Ruda Milicka	RRL	PelSAI-1 of parents	32	F	FFPE	TUNEL
**IF frozen**										
800/17/14	LL	Urwitałt	RR	Ruda Milicka	RL	PelSAI-1	41	F	PFA-fro	Nup-WGA
1615/3/15	RR	Ruda Milicka	LL	Raków	RL	PelSAI-1	41	F	PFA-fro	Nup-WGA
1616/3/15	RR	Ruda Milicka	LL	Raków	RL	PelSAI-1	44	F	PFA-fro	Nup, Cas3
1665/2/15	RR	Ruda Milicka	LL	Raków	RL	PelSAI-1	38	M	PFA-fro	Nup-WGA
218/37/16	RR	Sanie	LL	Domasz.	RL	PelSAI-1	32	M	PFA-fro	Nup-Cas3
222/37/16	RR	Sanie	LL	Domasz.	RL	PelSAI-1	29	F	PFA-fro	Nup-WGA
225/41/16	RR	Sanie	LL	Domasz.	RL	PelSAI-1	34	F	PFA-fro	Nup-Cas3
229/41/16	RR	Sanie	LL	Domasz.	RL	PelSAI-1	28	M	PFA-fro	Nup-WGA
254/10/16	LL	Sanie	LL	Sanie	LL	PelSAI-1	30	M	PFA-fro	Nup-Cas3

Individual	F parent taxon	origin	M parent taxon	origin	tadpole takson	tadpole genotyping method	Gosner stage	Tadpole sex	gonad fix. method	analytical method
**IF frozen**										
257/37/16	RR	Sanie	LL	Domasz.	RL	PelSAI-1	32	M	PFA-fro	Nup-Cas3
259/37/16	RR	Sanie	LL	Domasz.	RL	PelSAI-1	31	F	PFA-fro	Nup-Cas3
283/29/16	RR	Sanie	RR	Sanie	RR	PelSAI-1	30	M	PFA-fro	Nup-Cas3
376/37/16	RR	Sanie	LL	Domasz.	RL	PelSAI-1	34	M	PFA-fro	Nup-Cas3
**Whole mount IF**										
1001/10/16	LL	Sanie	LL	Sanie	LL	FISH	30	M	PFA-WhM	H3k9me3
1002/10/16	LL	Sanie	LL	Sanie	LL	FISH	32	F	PFA-WhM	H3k9me4
1014/31/16	LL	Domasz.	RL	Wysoka Kamieńska	LL	FISH	29	M	PFA-WhM	H4ac
1016/31/16	LL	Domasz.	RL	Wysoka Kamieńska	LL	FISH	31	F	PFA-WhM	H4ac
1025/23/16	LL	Domasz.	RL	Wysoka Kamieńska	RL	FISH	31	M	PFA-WhM	H3k9me3
1026/23/16	LL	Domasz.	RL	Wysoka Kamieńska	RL	FISH	30	M	PFA-WhM	H3k9me3
1027/23/16	LL	Domasz.	RL	Wysoka Kamieńska	RL	FISH	34	M	PFA-WhM	H3k9me3
1034/11/16	RL	Uciechow	LL	Domasz.	RL	FISH	33	F	PFA-WhM	H3k9me4
1035/11/16	RL	Uciechow	LL	Domasz.	RL	FISH	32	M	PFA-WhM	H3k9me5
1036/11/16	RL	Uciechow	LL	Domasz.	RL	FISH	31	F	PFA-WhM	H3k9me6
1037/11/16	RL	Uciechow	LL	Domasz.	RL	FISH	34	F	PFA-WhM	H4ac
1038/11/16	RL	Uciechow	LL	Domasz.	RL	FISH	30	F	PFA-WhM	H4ac
1039/11/16	RL	Uciechow	LL	Domasz.	RL	FISH	32	M	PFA-WhM	H4ac
1042/4/16	RL	Wysoka Kamieńska	RLL	Wysoka Kamieńska	RLL	FISH	31	M	PFA-WhM	H3k9me3
1043/4/16	RL	Wysoka Kamieńska	RLL	Wysoka Kamieńska	RLL	FISH	33	F	PFA-WhM	H3k9me3
**TEM**										
363/4/16	RL	Wysoka Kamieńska	RLL	Wysoka Kamieńska	RLL	Microsat.	36	M	Karnov.	TEM
364/4/16	RL	Wysoka Kamieńska	RLL	Wysoka Kamieńska	RLL	Microsat.	34	F	Karnov.	TEM
365/4/16	RL	Wysoka Kamieńska	RLL	Wysoka Kamieńska	RLL	Microsat.	34	M	Karnov.	TEM
366/2/16	RL	Uciechow	LL	Sanie	RL	Microsat.	35	F	Karnov.	TEM
367/2/16	RL	Uciechow	LL	Sanie	RL	Microsat.	29	M	Karnov.	TEM
368/2/16	RL	Uciechow	LL	Sanie	RL	Microsat.	32	F	Karnov.	TEM
012/07	RR	Ruda Milicka	RL	Ruda Milicka	RL	AMD-DAPI	38	M	Karnov.	TEM
348/07	RR	Ruda Milicka	LL	Raków	RL	AMD-DAPI	46	M	Karnov.	TEM
312/07	RR	Ruda Milicka	LL	Raków	RL	AMD-DAPI	46	M	Karnov.	TEM
130/07	RR	Ruda Milicka	LL	Raków	RL	AMD-DAPI	46	M	Karnov.	TEM
100/88	RL	Kotowice	LL	Kotowice	RL	Morphol.	36	F	Karnov.	TEM
128/88	RL	Kotowice	LL	Kotowice	RL	Morphol.	41	F	Karnov.	TEM
70/88	RR	Poznań	LL	Poznań	RL	Morphol.	34	F	Karnov.	TEM
71/88	LL	Poznań	RR	Poznań	RL	Morphol.	34	M	Karnov.	TEM
72/88	RR	Poznań	LL	Poznań	RL	Morphol.	31	F	Karnov.	TEM

Individual	F parent taxon	origin	M parent taxon	origin	tadpole takson	tadpole genotyping method	Gosner stage	Tadpole sex	gonad fix. method	analytical method
**TEM**										
79/88	RR	Poznań	LL	Poznań	RL	Morphol.	35	F	Karnov.	TEM
80/88	RL	Kotowice	LL	Kotowice	RL	Morphol.	36	F	Karnov.	TEM
81/88	RL	Kotowice	LL	Kotowice	RL	Morphol.	27	F	Karnov.	TEM
85/88	RL	Kotowice	LL	Kotowice	RL	Morphol.	29	F	Karnov.	TEM
87/88	RL	Kotowice	LL	Kotowice	RL	Morphol.	37	M	Karnov.	TEM
89/88	LL	Poznań	RR	Poznań	RL	Morphol.	40	F	Karnov.	TEM
90/88	RR	Poznań	LL	Poznań	RL	Morphol.	40	F	Karnov.	TEM
95/88	RR	Poznań	LL	Poznań	RL	Morphol.	40	F	Karnov.	TEM

References

1. Hauswaldt, J. S. *et al.* A simplified molecular method for distinguishing among species and ploidy levels in European water frogs (Pelophylax). *Mol. Ecol. Resour.* **12,** 797–805 (2012).
2. Arioli, M. Reproductive patterns and population genetics in pure hybridogenetic water frog populations of Rana esculenta. (University of Zurich, 2007).
3. Christiansen, D. G. Gamete types, sex determination and stable equilibria of all-hybrid populations of diploid and triploid edible frogs (Pelophylax esculentus). *BMC Evol. Biol.* **9,** 135 (2009).
4. Hotz, H. *et al.* Microsatellites: A tool for evolutionary genetic studies of western Palearctic water frogs. *Zoosystematics Evol.* **77,** 43–50 (2001).
5. Garner, T. W., Gautschi, B., Röthlisberger, S. & Reyer, H. U. A set of CA repeat microsatellite markers derived from the pool frog, Rana lessonae. *Mol. Ecol.* **9,** 2173–2175 (2000).

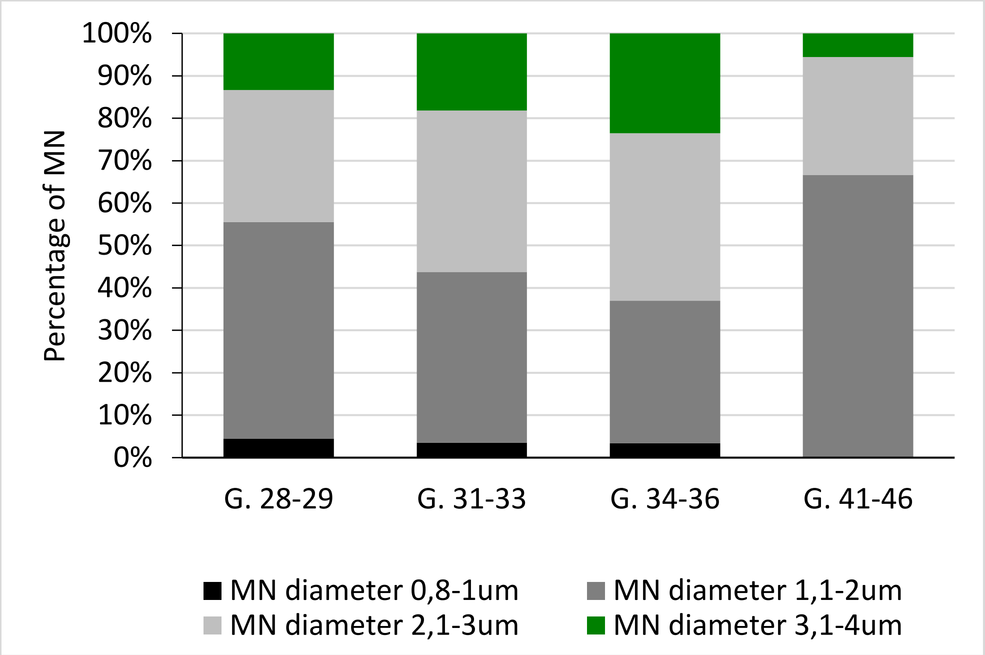

Supplementary Figure S1. Frequency of various micronuclei sizes in gonocytes of hybrid frogs during development according to Gosner stages (G)

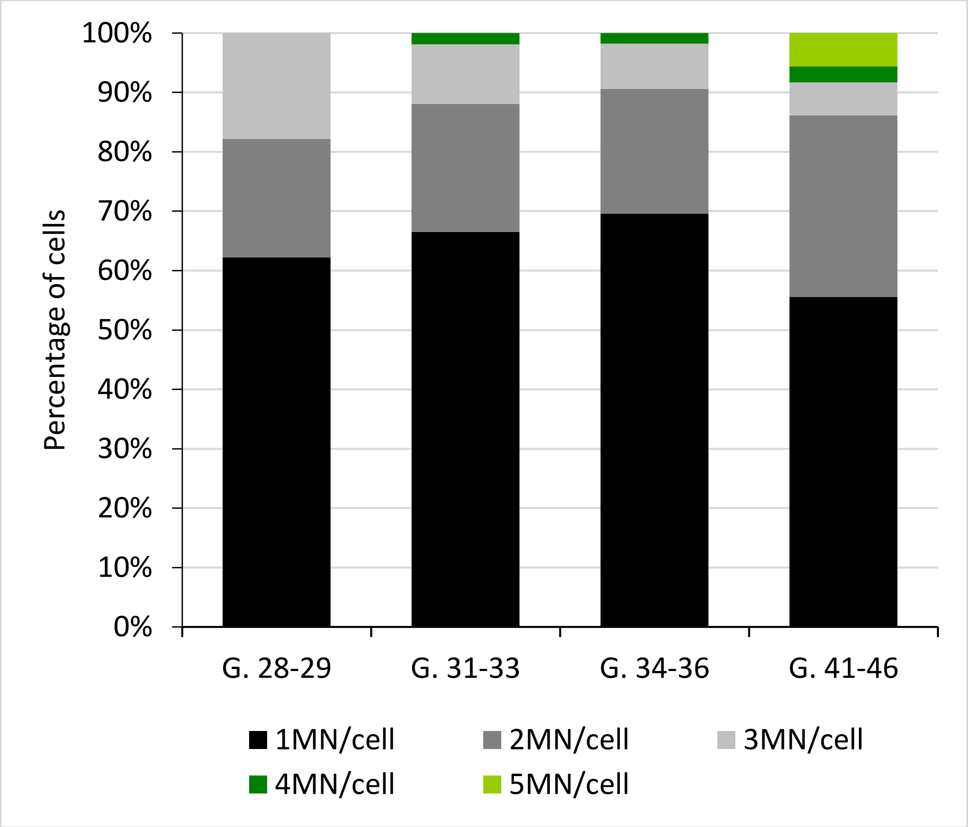

Supplementary Figure S2. Frequency of gonocytes hosting various numbers of micronuclei (MN) during development according to Gosner stages (G)

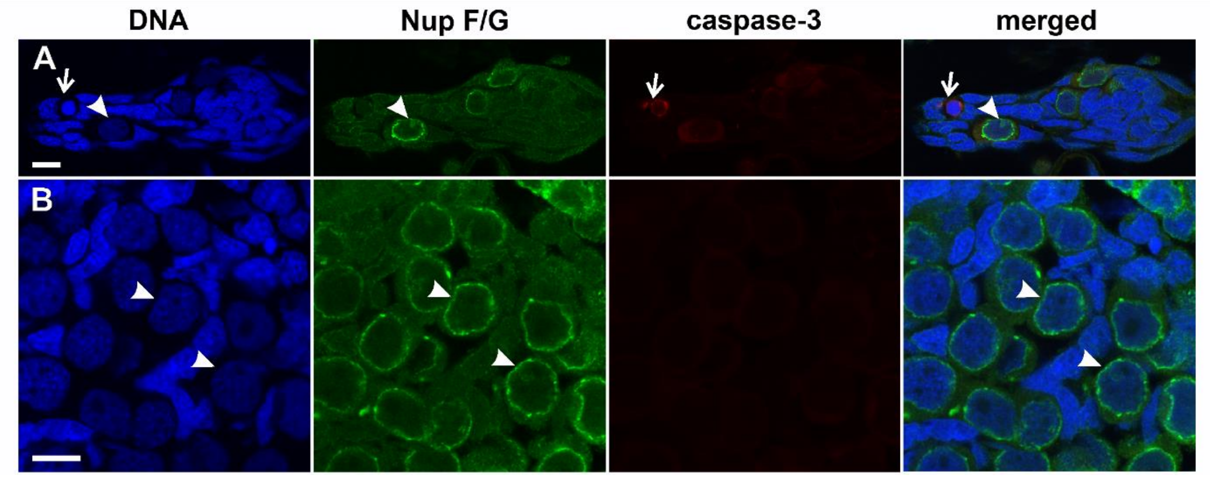

Supplementary Figure S3. The levels of nuclear pore complex proteins (Nup) and the frequency of apoptosis in gonocytes of parental species

Immunofluorescent staining of frozen tissue sections of gonads from *P. lessonae* (A. B) male at 30 Gosner stage: active caspase-3 (red), NPC proteins (Nup F/G. green), DNA counterstained with DAPI (blue). Gonocytes are characterized by weak chromatin staining corresponding mainly to euchromatin, whereas somatic cells have very strong chromatin staining due to heterochromatinization. (A) The distal part of shortening testis with apoptotic spermatogonium, note the pycnotic cell nucleus, lack of NPC proteins at the nuclear envelope and strong active caspase-3 signal in the cytoplasm. Normal germ cell is showed by arrowhead. (B) Prespermatogonia in the proximal part of developing testis do not show the apoptosis signs and have a very strong expression of NPC proteins (arrowheads), stronger than somatic cells in differentiated testis (round nuclei with bright DAPI staining). Scale bars 10 µm.

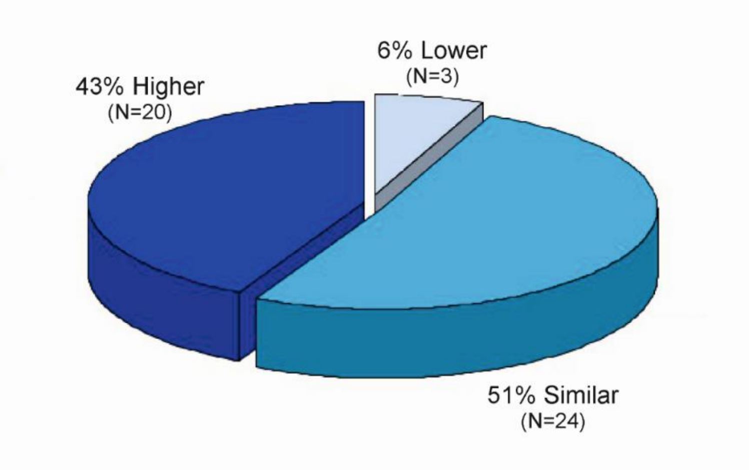

Supplementary Figure S4. Frequency of different heterochromatinization states in micronuclei in relation to main nuclei of gonocytes

To assess whether micronuclei possessed stronger DAPI signal (reflecting the DNA density) than main nuclei we calculated the ratio of micronucleus mean fluorescence value to main nucleus mean fluorescence value. Compared groups were chosen arbitrally in ranges: less than 0.79 – MN have lower fluorescence than nuclei; 0.8-1.2 – fluorescence levels are similar in MN and nuclei; higher than 1.21 – MN have distinctly higher DAPI signal than main nuclei.

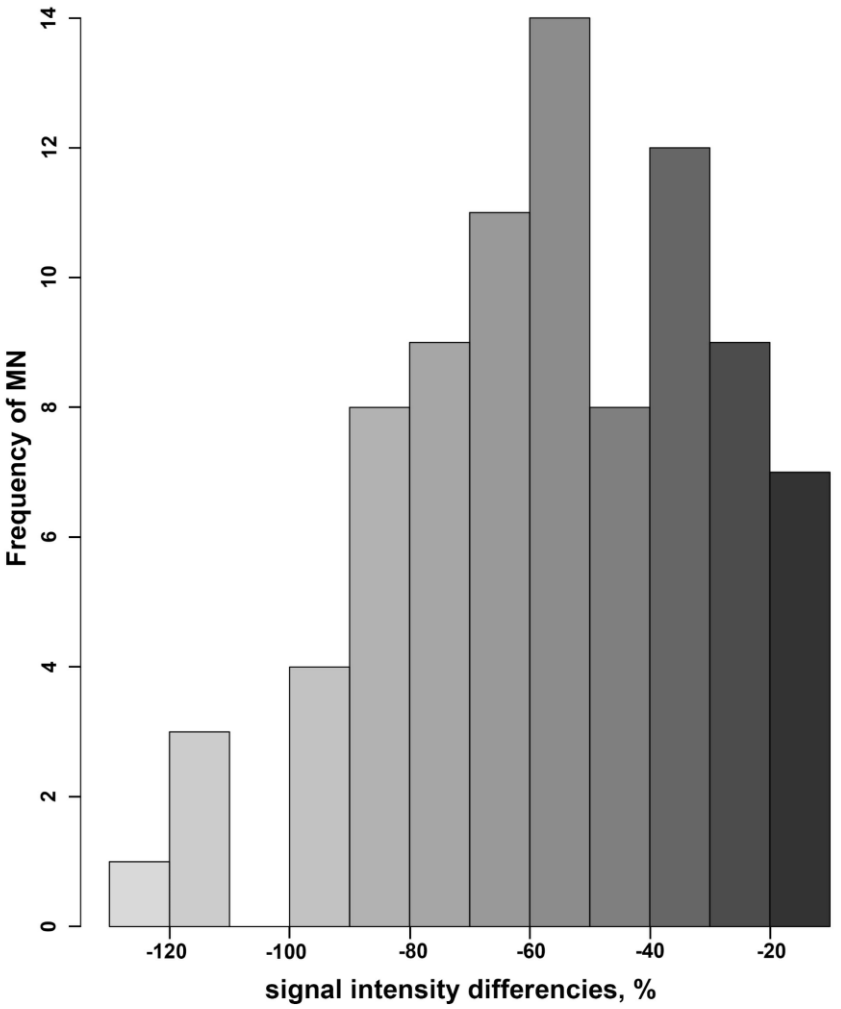

Supplementary Figure S5. Graph indicating comparison of H4Ac signal intensity in micronuclei (MN) comparatively to nuclei

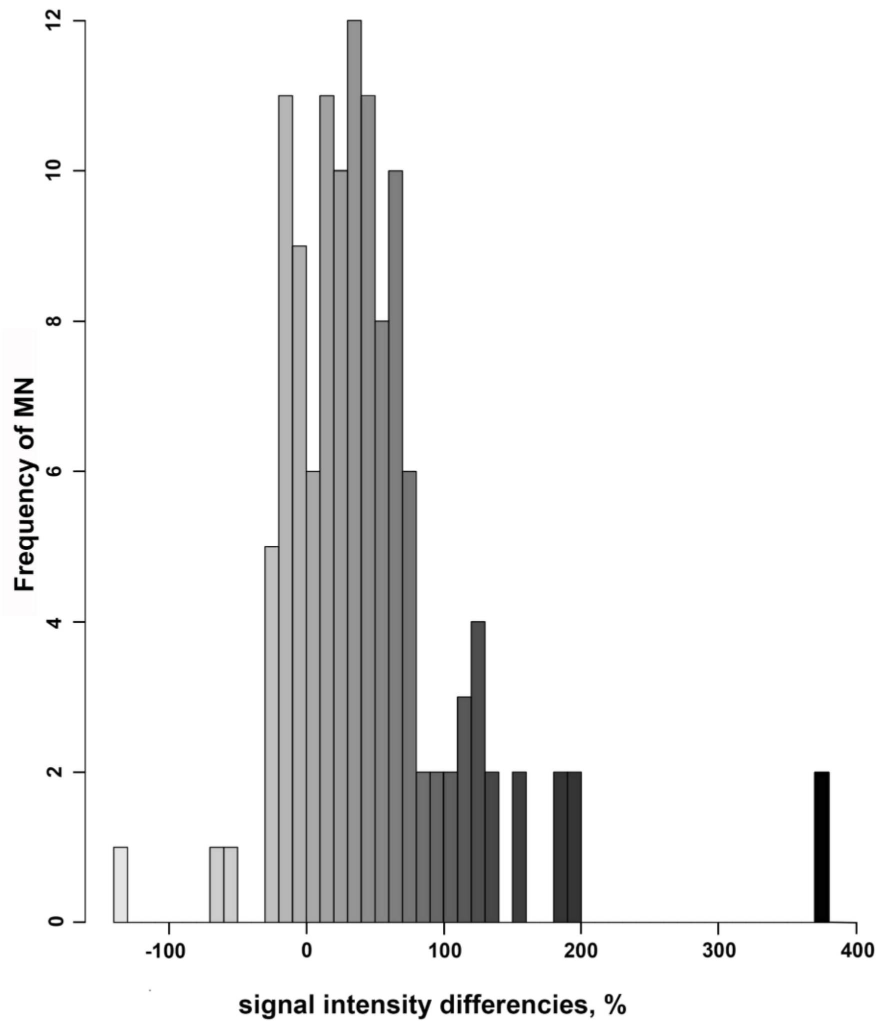

Supplementary Figure S6. Graphs indicating comparison of H3K9me3 signal intensity in micronuclei (MN) comparatively to nuclei

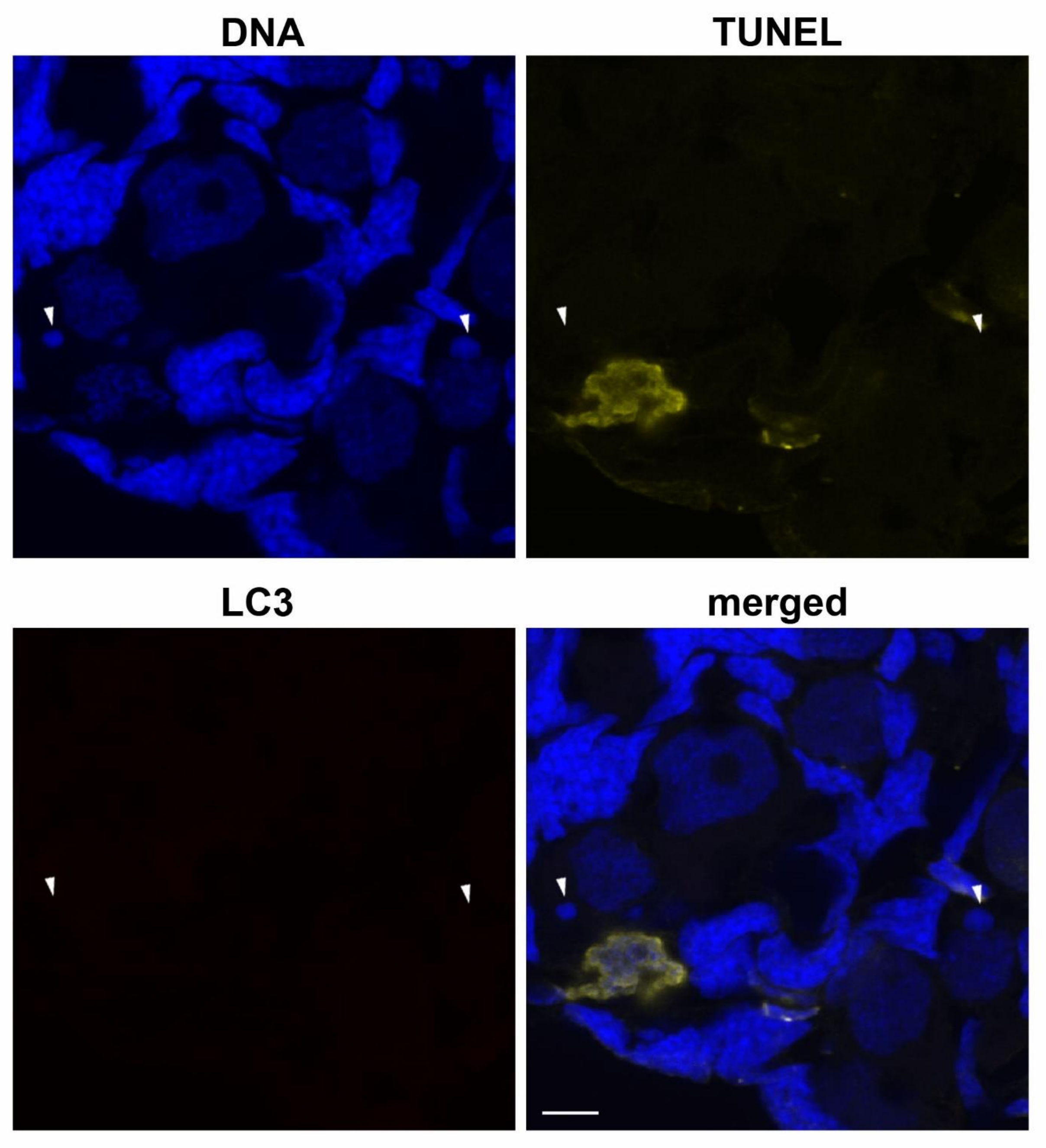

Supplementary Figure S7. Micronuclei do not accumulate double strand breaks in gonocytes of hybrid frog

Immunofluorescent detection of TUNEL reaction and LC3 staining of parafin tissue section of gonad from *P. esculentus* male at 28 Gosner stage: LC3 (red), TUNEL (yellow), DNA counterstained with DAPI (blue). Gonocytes are characterized by weak chromatin staining corresponding mainly to euchromatin, whereas somatic cells have very strong chromatin staining due to heterochromatinization. The cell on the left displays strong TUNEL signal according to apoptotic DNA degradation. Two micronuclei (arrowheads) inside two separate gonocytes show neither LC3 staining nor TUNEL signal. revealing that DNA is degraded in not apoptotic manner. Scale bar 5 μm.

Supplementary Movie S1. Early stage of micronucleus formation via nuclear budding

3D reconstruction of sequential confocal images of the gonocyte representing DNA (DAPI, blue) NPC proteins (Nup F/G, green) with nuclear bud and one separated micronucleus. Note the nuclear membrane fold visualised with NPC proteins surrounding the nuclear bud at the site of main nucleus and outside the nucleus. The protrusion of nuclear membrane emerging toward the separated micronucleus is visible which may represent recently detached micronucleus. Ovary of diploid *P. esculentus* female at Gosner stage 41.

Supplementary Movie S2. Later stage of micronucleus formation via nuclear budding

3D reconstruction of sequential confocal images of the gonocyte representing DNA (DAPI, blue) and NPC proteins (Nup F/G, green) with finger-like chromatin bud and one separated micronucleus. Note that micronucleus and a chromatin bud are lacking nuclear membrane. NPC proteins are present in the nuclear membrane of the main nucleus in the vicinity of separated micronucleus. Ovary of diploid *P. esculentus* female at Gosner stage 44.
